# STIM-mediated calcium influx regulates maintenance and selection of germinal center B cells

**DOI:** 10.1084/jem.20222178

**Published:** 2023-10-30

**Authors:** Yutaro Yada, Masanori Matsumoto, Takeshi Inoue, Akemi Baba, Ryota Higuchi, Chie Kawai, Masashi Yanagisawa, Daisuke Kitamura, Shouichi Ohga, Tomohiro Kurosaki, Yoshihiro Baba

**Affiliations:** 1 Division of Immunology and Genome Biology, Medical Institute of Bioregulation, Kyushu University, Fukuoka, Japan; 2 Department of Pediatrics, Graduate School of Medical Sciences, Kyushu University, Fukuoka, Japan; 3 Laboratory of Lymphocyte Differentiation, WPI Immunology Frontier Research Center, Osaka University, Osaka, Japan; 4 Department of Pathobiology, University of Illinois at Urbana-Champaign, Urbana, IL, USA; 5 International Institute for Integrative Sleep Medicine (WPI-IIIS), University of Tsukuba, Tsukuba, Japan; 6 Research Institute for Biomedical Sciences, Tokyo University of Science, Chiba, Japan; 7 Laboratory for Lymphocyte Differentiation, RIKEN Center for Integrative Medical Sciences, Yokohama, Japan

## Abstract

Positive selection of high-affinity germinal center (GC) B cells is driven by antigen internalization through their B cell receptor (BCR) and presentation to follicular helper T cells. However, the requirements of BCR signaling in GC B cells remain poorly understood. Store-operated Ca^2+^ entry, mediated by stromal interacting molecule 1 (STIM1) and STIM2, is the main Ca^2+^ influx pathway triggered by BCR engagement. Here, we showed that STIM-deficient B cells have reduced B cell competitiveness compared with wild-type B cells during GC responses. B cell–specific deletion of STIM proteins decreased the number of high-affinity B cells in the late phase of GC formation. STIM deficiency did not affect GC B cell proliferation and antigen presentation but led to the enhancement of apoptosis due to the impaired upregulation of anti-apoptotic Bcl2a1. STIM-mediated activation of NFAT was required for the expression of Bcl2a1 after BCR stimulation. These findings suggest that STIM-mediated survival signals after antigen capture regulate the optimal selection and maintenance of GC B cells.

## Teaser

STIM-dependent calcium signaling after antigen recognition promotes GC B cell survival, leading to the maintenance and selection of high-affinity B cells during the GC reaction.

## Graphical Abstract

**Figure:**
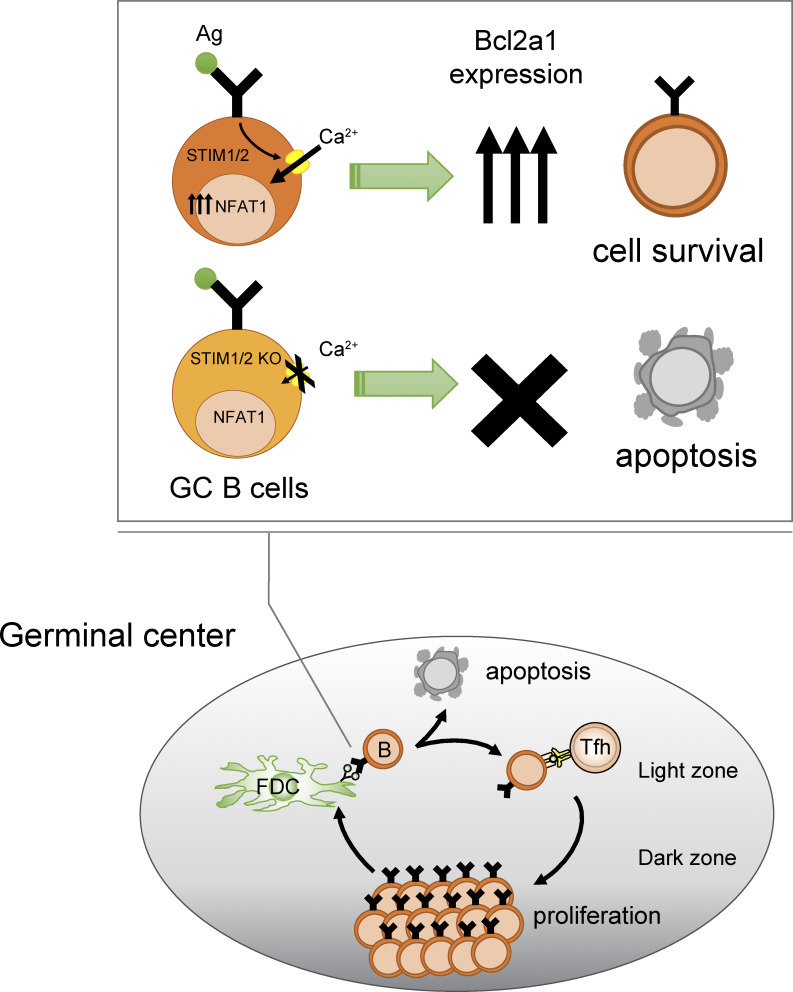

## Introduction

Germinal centers (GCs) are specialized microenvironments where antigen (Ag)-specific B cells undergo antibody affinity maturation and clonal expansion (De Silva and Klein, 2015; Ise and Kurosaki, 2019; Laidlaw and Cyster, 2021; Rajewsky, 1996; Victora and Nussenzweig, 2022; Vinuesa et al., 2016). B cells bearing high-affinity antibodies give rise to plasma cells that are integral to the humoral immune response. GCs are comprised of two distinct zones: a dark zone (DZ), where B cells undergo clonal expansion and somatic hypermutation (SHM) in the immunoglobulin gene, and a light zone (LZ), where B cells compete to capture Ags on follicular dendritic cells (FDCs) and then present these to follicular helper T (Tfh) cells (Bannard and Cyster, 2017; Ise and Kurosaki, 2019; Laidlaw and Cyster, 2021). The positive selection of GC B cells requires that they recognize the Ag via the B cell receptor (BCR) and obtain signals from Tfh cells. GC B cells internalize Ags for presentation to a limited number of Tfh cells, which favors B cells with the highest density of Ag-derived peptide-loaded major histocompatibility complexes (pMHCs) on their surface with the highest BCR affinity (Allen et al., 2007; Liu et al., 2015; Victora et al., 2010; Vinuesa and Cyster, 2011). When B cells receive Tfh critical signals, they are actively selected; otherwise, they are eliminated by apoptosis (Ise and Kurosaki, 2019; Laidlaw and Cyster, 2021; Mayer et al., 2017; Victora and Nussenzweig, 2022). Successful B cell competitors re-enter the DZ to continue proliferation and SHM, thus maximizing the somatic evolution of BCR affinity (Gitlin et al., 2014; Ise and Kurosaki, 2019; Laidlaw and Cyster, 2021; Victora and Nussenzweig, 2022). Other mechanisms of affinity-driven selection have also been proposed, including (1) affinity-dependent apoptosis in the LZ (high-affinity B cells are allowed to reenter the DZ, but low-affinity B cells are eliminated by apoptosis) and (2) affinity-dependent proliferation in the DZ (GC B cell growth is dependent on their affinity) (Amitai et al., 2017; Victora and Nussenzweig, 2022). Several studies have elucidated the signaling pathway for the positive selection of GC B cells. For example, CD40 signals from Tfh cells are required for GC initiation and maintenance, which cannot be induced by BCR stimulation alone, but simultaneous BCR and CD40 ligation synergistically induces the selective proliferation of high-affinity GC B cells (Luo et al., 2018). However, the precise role of affinity-dependent BCR signaling in GC B cells remains unclear.

Ag-specific immune responses require BCR signaling whose major event is an increase in calcium ion (Ca^2+^) concentrations in the cytosol (Kurosaki et al., 2010). Although BCR signals in GC B cells were thought to be attenuated (Khalil et al., 2012), purified GC B cells evoked Ca^2+^ fluxes that were similar to or slightly higher than those in naive B cells when stimulated with Ags in vitro (Nowosad et al., 2016). In addition, in vivo two-photon fluorescence imaging revealed elevated Ca^2+^ signals in Ag-specific B cells when in contact with FDCs in GCs (Ulbricht et al., 2021). The chief source of Ca^2+^ signals in B cells is store-operated Ca^2+^ entry (SOCE) through Ca^2+^ release-activated channels, which induces a sustained influx of extracellular Ca^2+^ (Baba and Kurosaki, 2011). BCR engagement triggers the production of inositol-1,4,5-trisphosphate (IP_3_) and releases Ca^2+^ from the endoplasmic reticulum (ER) Ca^2+^ store. Stromal interacting molecule 1 (STIM1) and STIM2 are calcium sensors within the ER that couple the reduction of ER Ca^2+^ stores to the plasma membrane Orai channel (Baba et al., 2014; Feske, 2007; Liou et al., 2005; Roos et al., 2005). Using mice lacking STIM1 and STIM2 in B cells (*Stim1/2* BKO), we previously showed that BCR-mediated Ca^2+^ is critical for interleukin-10 (IL-10) production through the calcineurin/nuclear factor of activated T cells (NFAT) pathway, which limits autoimmune inflammation (Baba and Kurosaki, 2011; Matsumoto et al., 2011). Although *Stim1/2* BKO mice appear to show normal antibody responses (Matsumoto et al., 2011), the detailed responses of GC B cells are poorly understood.

Here, we have explored the role of BCR-mediated SOCE in GC responses. We found that, in chimeric mice populated by STIM-sufficient and -deficient B cells, the affinity maturation of GC B cells required STIM proteins. In the absence of STIM proteins, high-affinity GC B cells showed poor survival rates due to impaired induction of the anti-apoptotic B cell lymphoma 2–related protein a1 (*Bcl2a1*) gene, which is required for NFAT activation dependent on STIM-mediated increases in Ca^2+^ levels. Our study provides empirical evidence for the importance of STIM-dependent Ca^2+^ signaling in GC B cell maintenance and selection.

## Results

### STIM deficiency decreases GC B cell competitiveness

**Figure 1.**
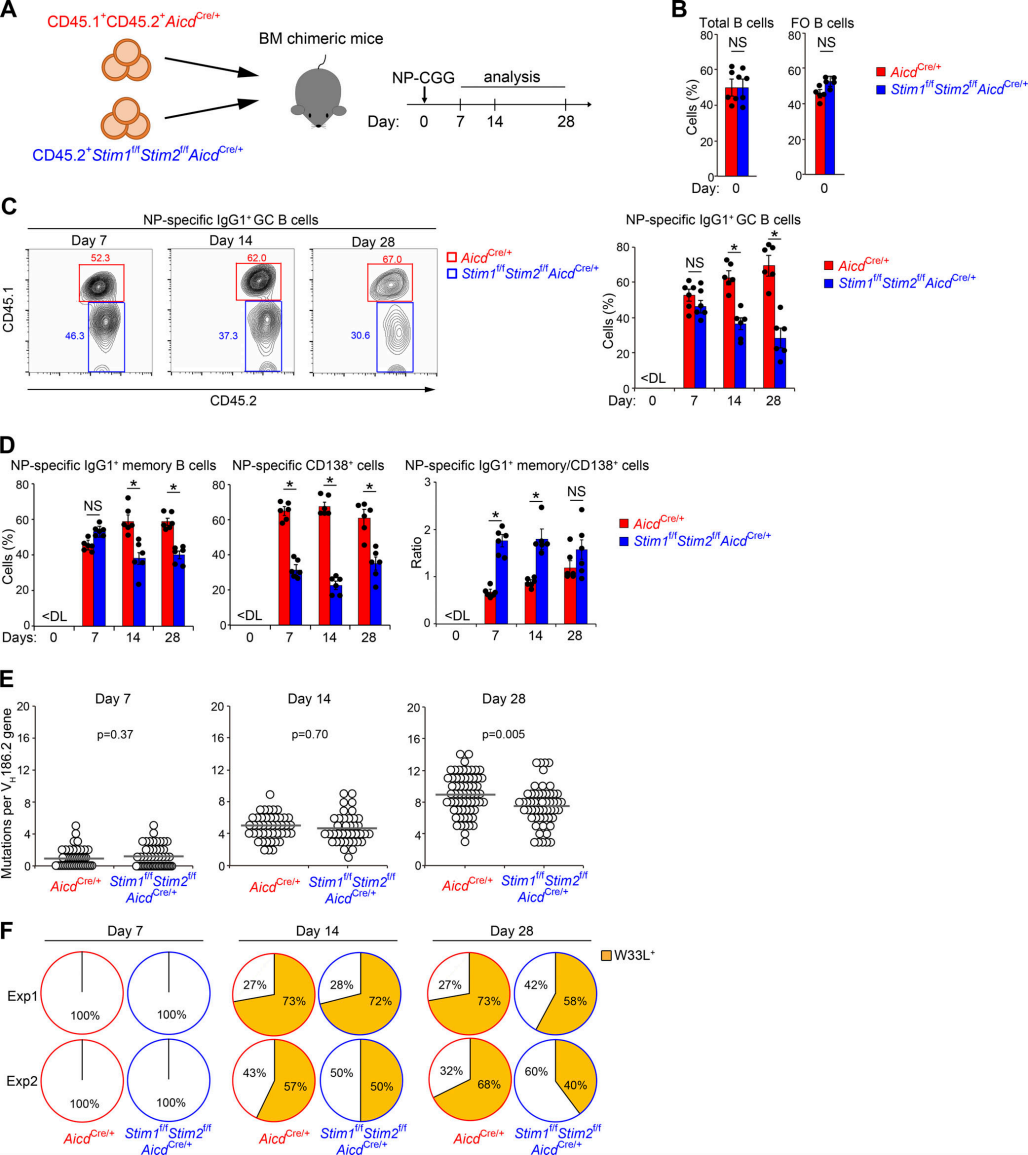
**STIM proteins are required for effective competition in GC. (A)** Schematic of experimental workflow. Mixed BM chimeric mice (μMT mice lethally irradiated and reconstituted with 50% CD45.1^+^CD45.2^+^*Aicd*^*Cre*/+^ plus 50% CD45.2^+^*Stim*1^f/f^*Stim2*^f/f^*Aicd*^*Cre*/+^ BM cells) were immunized with NP-CGG in alum. On the indicated time point (day 7∼28), the mice were sacrificed and analyzed. **(B–D)** Frequency of CD45.1^+^CD45.2^+^*Aicd*^*Cre*/+^ and CD45.2^+^*Stim*1^f/f^*Stim2*^f/f^*Aicd*^*Cre*/+^ cells in indicated parental populations in the spleen of mixed BM chimeric mice before (B) and after immunization with NP-CGG in alum (C and D). Total B, follicular (FO) B, and NP-specific IgG1^+^ GC, IgG1^+^ memory B, and CD138^+^ cells are defined as B220^+^, CD21^low^CD23^high^B220^+^, IgG1^+^NIP^+^CD38^low^B220^+^, IgG1^+^NIP^+^CD38^high^B220^+^, and NIP^+^CD138^+^B220^low^ cells, respectively. Data are presented as mean ± SEM for five or six mice. Data are representative of two independent experiments. NS, not significant. *, P < 0.05 versus *Aicd*^*Cre*/+^ cells ([B] Student’s *t* test and [C and D] two-way ANOVA). **(E)** Accumulation of mutations in *V*_*H*_*186.2* genes of single NP-specific IgG1^+^ GC B cells in mixed BM chimeric mice immunized with NP-CGG in alum. Circles represent the number of mutations in individual clones. The results were evaluated statistically by Student’s *t* test. Data are representative of two independent experiments. **(F)** Frequency of W33L^+^ clones among NP-specific IgG1^+^ GC B cells in mixed BM chimeric mice immunized with NP-CGG in two separate experiments (Exp1 and Exp2). Numbers along the perimeter indicate percentages of W33L^−^ (white) and W33L^+^ (orange) clones.

**Figure S1.**
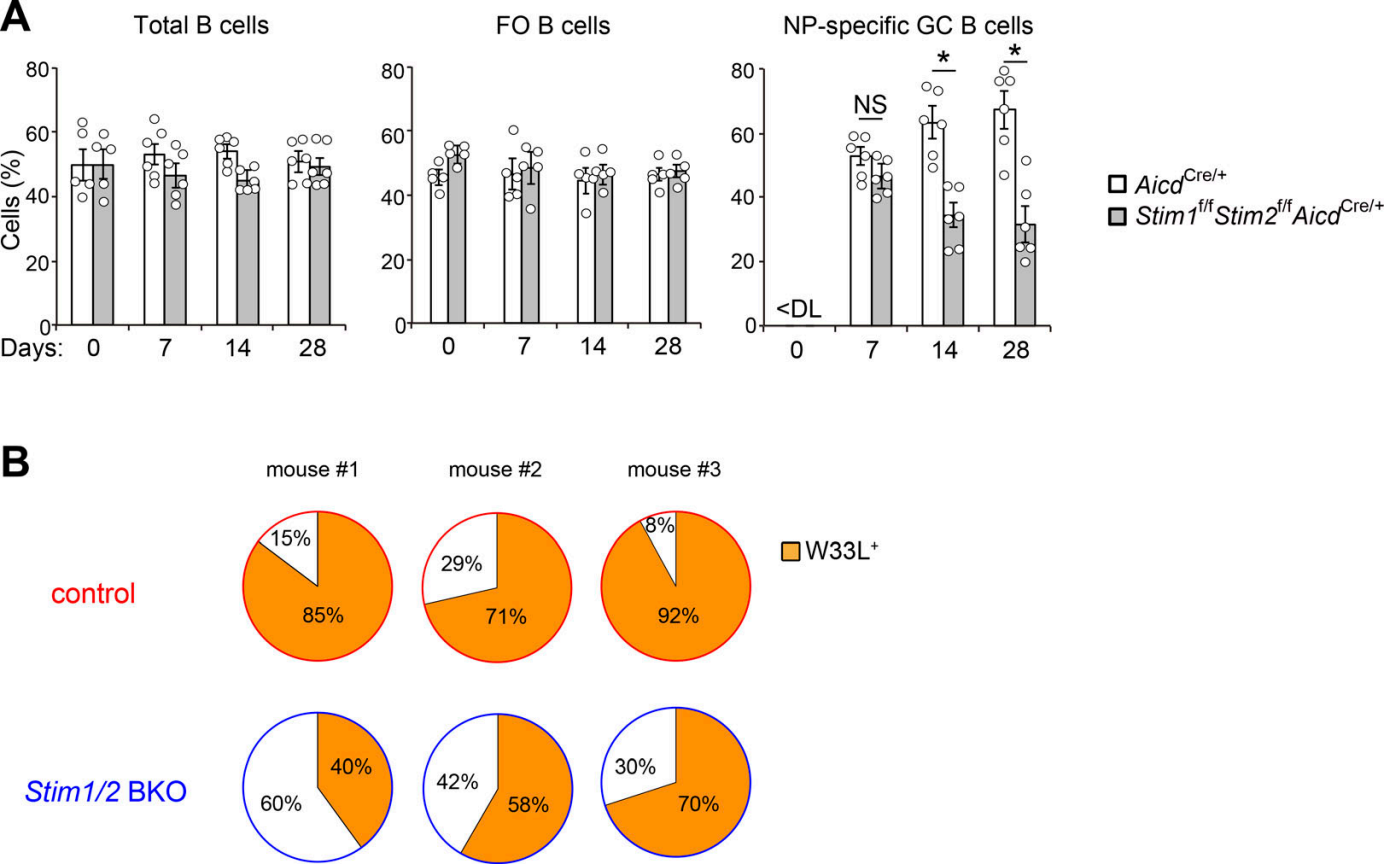
**STIM proteins are required for effective competition in GC.** Related to Fig. 1. **(A)** Frequency of CD45.1^+^CD45.2^+^*Aicd*^*Cre*/+^ and CD45.2^+^*Stim1*^f/f^*Stim2*^f/f^*Aicd*^*Cre*/+^ cells in indicated parental populations in the spleen of mixed BM chimeric mice immunized with NP-CGG in alum. Total B, follicular (FO) B, and NP-specific GC B cells are defined as B220^+^, CD21^low^CD23^high^B220^+^, and NIP^+^FAS^+^GL7^+^B220^+^ cells, respectively. Data are presented as mean ± SEM for five or six mice. Data are representative of two independent experiments. NS, not significant. *, P < 0.05 versus *Aicd*^*Cre*/+^ cells (two-way ANOVA). **(B)** Mixed BM chimeric mice (CD45.1^+^ mice lethally irradiated and reconstituted with 50% CD45.1^+^CD45.2^+^*Mb1*^*Cre*/+^ (control) plus 50% CD45.2^+^*Stim1*^f/f^*Stim2*^f/f^*Mb1*^*Cre*/+^ (*Stim1/2* BKO) BM cells) were immunized with NP-CGG in alum. Frequency of W33L^+^ clones among single NP-specific IgG1^+^ plasma cells (NIP^+^CD138^+^TACI^+^IgG1^+^) in mixed BM chimeric mice immunized with NP-CGG for 14 days. Numbers along the perimeter indicate percentages of W33L^−^ (white) and W33L^+^ (orange) clones. Data are representative of two independent experiments.

To examine whether STIM proteins are essential for GC B cell responses in a competitive setting, we generated mixed bone marrow (BM) chimeric mice by transferring equal numbers of BM cells from CD45.1^+^CD45.2^+^*Aicd*^*Cre*/+^ and CD45.2^+^*Stim1*^f/f^*Stim2*^f/f^*Aicd*^*Cre*/+^ mice into lethally irradiated B cell–deficient μMT mice (Fig. 1 A). The frequency of total and follicular *Stim1*^f/f^*Stim2*^f/f^*Aicd*^*Cre*/+^ B cells in the spleen of chimeric mice was comparable with that of control *Aicd*^*Cre*/+^ cells in unimmunized mice (Fig. 1 B). To determine the effect of STIM proteins on Ag-specific GC responses, chimeric mice were immunized with a T cell–dependent Ag composed of 4-hydroxy-3-nitrophenyl-chicken γ globulin (NP-CGG) in alum adjuvant. On day 7 after immunization, the percentages of *Aicd*^*Cre*/+^ and *Stim1*^f/f^*Stim2*^f/f^*Aicd*^*Cre*/+^ B cells among NP-specific GC B cells were comparable (Fig. 1 C and Fig. S1 A). However, NP-specific STIM-deficient GC B cells were gradually outcompeted by control cells and their percentages were decreased by ∼50% 28 days after immunization (Fig. 1 C and Fig. S1 A). B cells leaving the GC become memory B or plasma cells (De Silva and Klein, 2015; Ise and Kurosaki, 2019; Laidlaw and Cyster, 2021; Victora and Nussenzweig, 2022). Thus, one possible reason for this reduction is due to an accelerated transit of these cells to the memory B or plasma cell compartment in the absence of STIM proteins. However, it is not likely because the populations of NP-specific memory B cells and CD138^+^ plasmablasts/plasma cells were also reduced in the absence of STIM proteins (Fig. 1 D). These results suggest that STIM proteins are critical for the effective generation and/or survival of GC B cells. In addition, when we examined the proportion of memory B cells and plasma cells, the ratio of memory B cells to plasma cells was higher in STIM-deficient cells in the early phase of the GC reaction (Fig. 1 D). Given that high-affinity B cells become plasma cells and low-affinity B cells become memory B cells, the loss of STIMs is likely to affect high-affinity GC B cells and plasma cell differentiation.

Consequently, we considered whether STIM proteins affect the SHMs of Ig variable regions in GC B cells. To determine whether STIM deficiency reduces affinity maturation, we performed polymerase chain reaction (PCR) on sorted GC B cells to evaluate the frequency of high-affinity mutations in the canonical NP-responding gene *V*_*H*_*186.2* at days 7, 14, and 28. Sequence analysis revealed that the number of somatic mutations in *V*_*H*_*186.2* of control cells accumulated continuously after immunization with NP-CGG (Fig. 1 E), consistent with the results of a previous report (Takahashi et al., 2001). In contrast, the number of mutations in STIM-deficient GC B cells was significantly lower than that in the control cells on day 28 after immunization (Fig. 1 E). The change of tryptophan to leucine at the 33rd amino acid position of the *V*_*H*_*186.2* gene (W33L) results in an ∼10-fold increase in BCR affinity for NP (Allen et al., 1988). We found that the W33L mutation in STIM-deficient GC B cells was reduced by ∼20% compared with that in control cells on day 28 after immunization (Fig. 1 F). Loss of STIM proteins also reduced the W33L mutation in antibody-secreting cells (Fig. S1 B). These observations suggest that STIM proteins contribute to the regulation of high-affinity GC B cell selection.

### STIM-mediated SOCE is essential for high-affinity GC B cell maintenance

**Figure 2.**
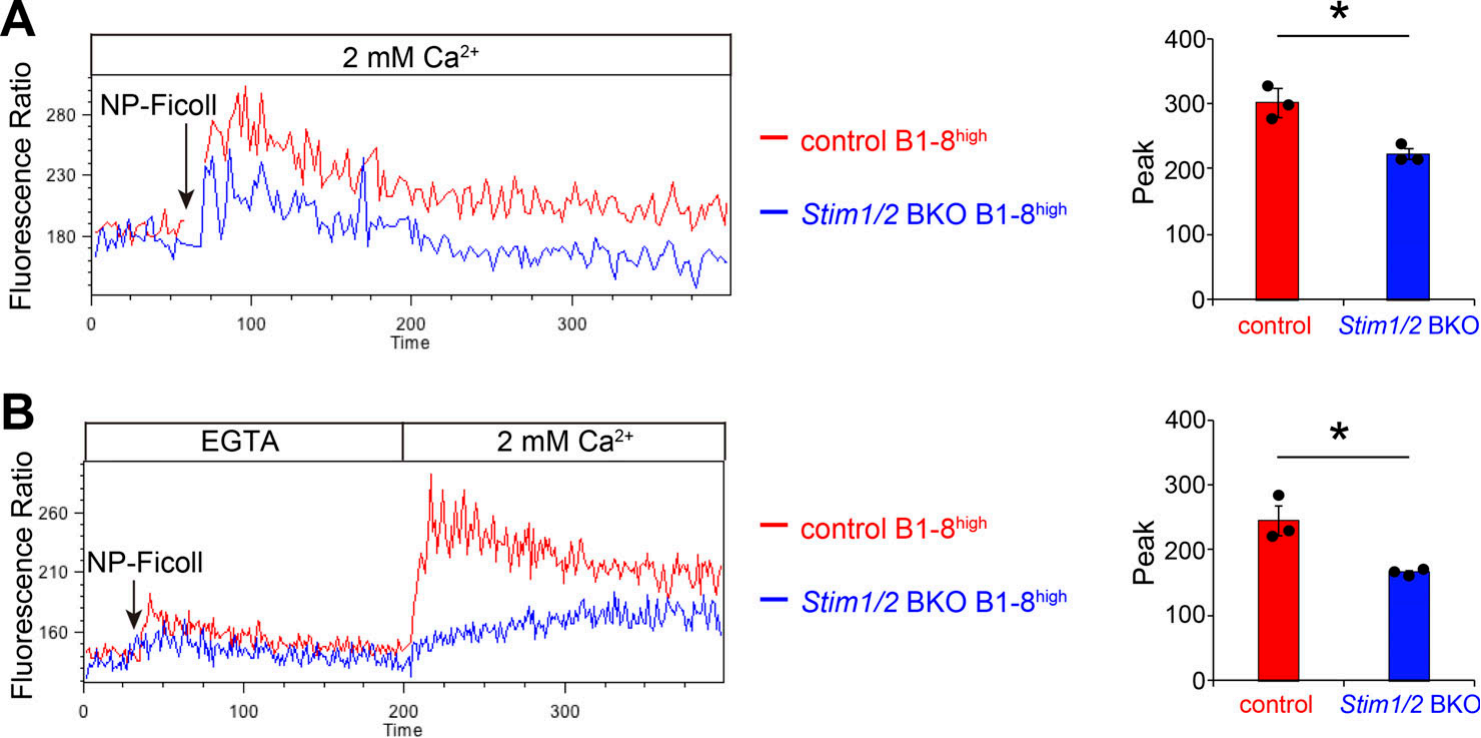
**STIM proteins are required for Ag-derived SOC influx. (A)** Ca^2+^-mobilization profiles monitored by Indo-1 AM imaging in the presence of 2 mM extracellular Ca^2+^ in *Mb1*^*Cre*/+^(control) B1-8^high^ and *Stim1*^f/f^*Stim2*^f/f^*Mb1*^*Cre*/+^(*Stim1/2* BKO) B1-8^high^ B cells after stimulation with NP-Ficoll. **(B)** Ca^2+^ release was elicited in control and *Stim1/2* BKO B1-8^high^ B cells by stimulation with NP-Ficoll in Ca^2+^-free conditions (0.5 mM EGTA), and Ca^2+^ influx was induced by restoration of the extracellular Ca^2+^ concentration to 2 mM. **(A and B)** B220^+^Igκ^−^ cells were gated and analyzed. All values are plotted as the FL5/FL4 fluorescence ratio (FL4 = 500–520 nm; FL5 = 400–420 nm). The peak of fluorescence ratios is shown on the right. Data are presented as mean ± SEM for three mice. Data are representative of two independent experiments. *, P < 0.05 versus control mice (Student’s *t* test).

To assess the effects of STIM proteins on SOCE in high-affinity B cells, we generated *Stim1/2* BKO B1-8^high^ mice with high-affinity B cells lacking STIM proteins by crossing *Stim1*^f/f^*Stim2*^f/f^*Mb1*^*Cre*/+^ with B1-8^high^ mice. The use of the *Mb1*^*Cre*/+^ driver allows us to assess the effect of STIM deficiency even in the early phase of the GC reaction. B1-8^high^ B cells had BCRs with a ten-fold higher affinity (Ka; 5 × 10^6^ M^−1^) because of a single W33L point mutation in the heavy chain (V_H_186.2) for NP hapten (Shih et al., 2002b). STIM deficiency in B1-8^high^ Igλ^+^ B cells led to reduced Ca^2+^ mobilization compared with that in control cells (*Mb1*^*Cre*/+^ B1-8^high^), and almost complete loss of SOCE after stimulation with NP-Ficoll Ag (Fig. 2, A and B), indicating that STIM proteins are essential for SOCE in high-affinity B cells.

**Figure 3.**
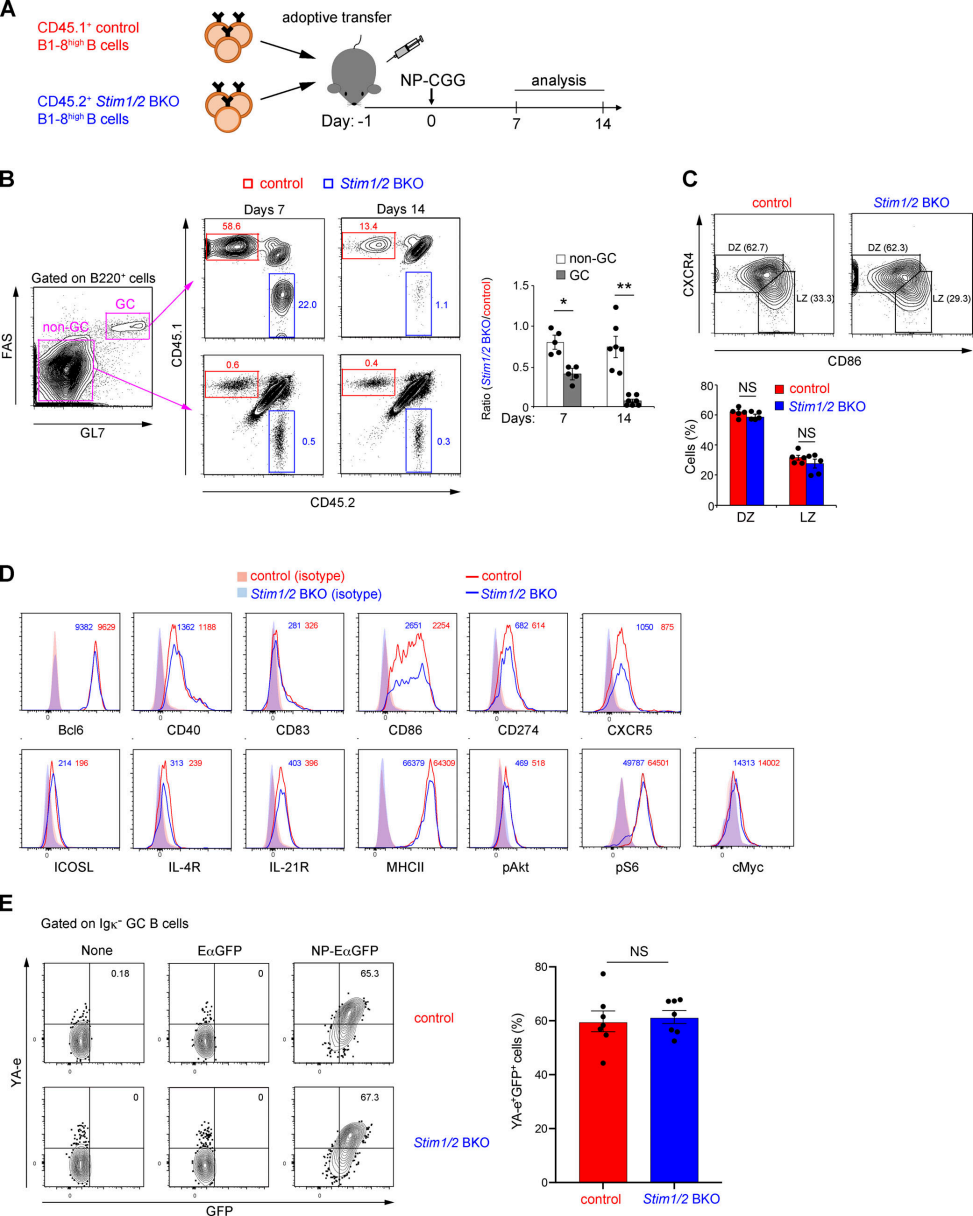
**GC B cell maintenance requires STIM-mediated SOC influx. (A)** Schematic of experimental workflow. CD45.1/2 wild-type mice transferred with equal number of CD45.1^+^ control B1-8^high^ and CD45.2^+^ *Stim1/2* BKO B1-8^high^ B cells were immunized with NP-CGG in alum. CD45.1/2 indicates cells from mice carrying both a CD45.1 and a CD45.2 allele. On the indicated time point (day 7 or 14), the mice were sacrificed and analyzed. **(B)** Flow cytometry of non-GC and GC B cells in the spleen of CD45.1/2 wild-type mice transferred with an equal number of CD45.1^+^ control B1-8^high^ and CD45.2^+^ *Stim1/2* BKO B1-8^high^ B cells, immunized with NP-CGG in alum for 7 or 14 days. Percentages of CD45.1^+^ control and CD45.2^+^ *Stim1/2* BKO cells in non-GC and GC B cells are shown. The ratios of CD45.2^+^ *Stim1/2* BKO B cells to CD45.1^+^ control B cells are shown on the right. Non-GC and GC B cells are defined as FAS^−^GL7^−^B220^+^ and FAS^+^GL7^+^B220^+^ cells, respectively. *, P < 0.05, **, P < 0.001 versus non-GC B cells (Student’s *t* test). **(C)** Flow cytometry of CD45.1^+^ control and CD45.2^+^ *Stim1/2* BKO GC B cells in spleen of CD45.1/2 wild-type mice transferred with equal number of CD45.1^+^ control B1-8^high^ and CD45.2^+^ *Stim1/2* BKO B1-8^high^ B cells, immunized with NP-CGG in alum for 7 days. The percentages of DZ and LZ GC B cells are shown at the bottom. DZ and LZ GC B cells are defined as CXCR4^high^CD86^low^CD38^low^GL7^+^ and CXCR4^low^CD86^high^CD38^low^GL7^+^ cells, respectively. NS, not significant (Student’s *t* test). **(D)** Flow cytometry of CD45.1^+^ control (red histogram) and CD45.2^+^ *Stim1/2* BKO GC B cells (blue histogram) in the spleen of CD45.1/2 wild-type mice transferred with equal number of CD45.1^+^ control B1-8^high^ and CD45.2^+^ *Stim1/2* BKO B1-8^high^ B cells, immunized with NP-CGG in alum for 7 days. Red and blue shaded curves indicate isotype control staining of GC B cells. Numbers adjacent to histograms indicate mean fluorescence intensity for each marker of GC B cells. **(E)** Flow cytometry of CD45.1^+^ control and CD45.2^+^ *Stim1/2* BKO GC B cells in spleen of CD45.1/2 wild-type mice transferred with equal number of CD45.1^+^ control B1-8^high^ and CD45.2^+^ *Stim1*/*2* BKO B1-8^high^ B cells immunized with NP-CGG in alum for 7 days. Purified CD43-negative B cells from splenocytes were incubated without or with EαGFP and NP-EαGFP for 1 h before Igκ^−^FAS^+^GL7^+^ cells were gated and analyzed. Percentages of YA-e^+^GFP^+^ B cells are shown on the right. NS, not significant (Student’s *t* test). **(B, C, and E)** Data are presented as mean ± SEM for six or seven mice. Data shown are pooled from at least three independent experiments. **(D)** Data are representative of two independent experiments (*n* = 4).

**Figure S2.**
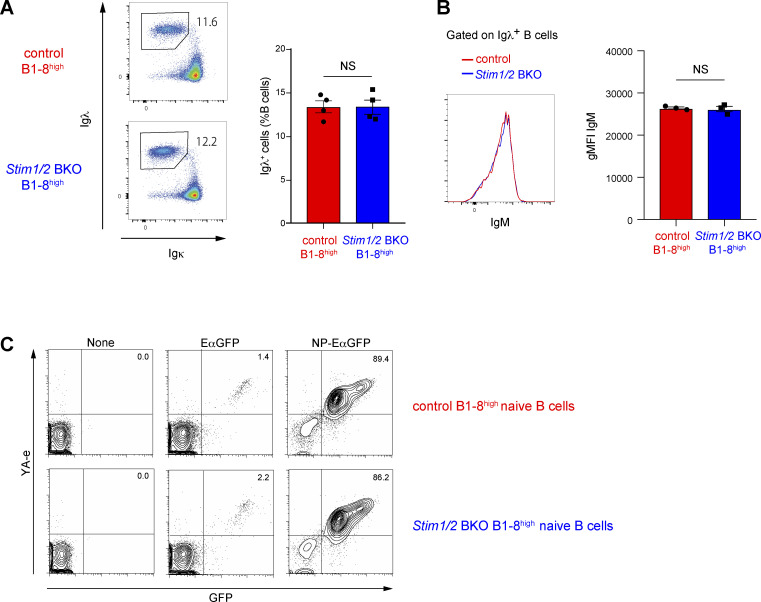
**Deficiency of SOC influx in B cells does not affect Igλ light chain usage, IgM expression, and Ag-presentation ability.** Related to Fig. 3. **(A and B)** Flow cytometry of control B1-8^high^ and *Stim1/2* BKO B1-8^high^ splenocytes. Percentages of Igλ^+^ B cells (A) and geometric mean fluorointensity (gMFI) of IgM in Igλ^+^ B cells (B) are shown. Data are representative of at least two independent experiments. NS, not significant (Student’s *t* test). **(C)** Flow cytometry of control B1-8^high^ and *Stim1/2* BKO B1-8^high^ naive B cells incubated without or with EαGFP and NP-EαGFP for 4 h. Percentages of YA-e^+^GFP^+^ B cells are shown. Data are representative of three independent experiments.

We assessed whether STIM-mediated SOCE is functionally important for the maintenance of high-affinity B cells in GCs. Accordingly, we transferred an equal number of splenic B cells isolated from CD45.1^+^ control B1-8^high^ and CD45.2^+^ *Stim1/2* BKO B1-8^high^ mice into congenic CD45.1/2 wild-type recipient mice 24 h before NP-CGG immunization (Fig. 3 A). Control B1-8^high^ and *Stim1/2* BKO B1-8^high^ mice had comparable percentages of NP-reactive Igλ^+^ cells and IgM expression among splenic B cells (Fig. S2, A and B). The ratio of STIM-deficient GC B cells to control cells was reduced by ∼60% and 90% at 7 and 14 days after immunization, respectively (Fig. 3 B). These results indicate that STIM-mediated SOCE is critical for the maintenance of high-affinity GC B cells.

GCs are separately localized in the DZ and LZ areas, where B cells undergo clonal expansion and present cognate Ags to Tfh cells, respectively (Bannard and Cyster, 2017; Kurosaki et al., 2015; Victora and Nussenzweig, 2022). Therefore, to test whether the decrease in the populations of STIM-deficient GC B cells is caused by the aberrant polarization of DZ and LZ B cells, we determined their percentages by measuring the expression of specific surface markers—CXC-chemokine receptor 4 (CXCR4) and CD86. We found that the loss of STIM proteins did not influence the frequency of DZ and LZ B cells (Fig. 3 C). Compared with control cells, STIM-deficient cells had similar intensities of GC B cell–associated molecules, including Bcl6, CD40, CD83, CD86, CD274 (PD-L1), CXCR5, inducible T cell co-stimulator ligand (ICOSL), IL-4 receptor (IL-4R), IL-21R, and major histocompatibility class II (MHC II) (Fig. 3 D). Although Akt activation is critical for GC B cells to avoid apoptosis (Green et al., 2011), STIM protein deficiency did not affect the expression of Akt phosphorylated at Thr308 (Fig. 3 D), which is critical for FOXO1 inactivation during GC B cell selection (Dominguez-Sola et al., 2015; Luo et al., 2018; Sander et al., 2015). The expression of c-Myc and the phosphorylation of S6 protein, which are regulated by BCR/CD40-mediated signals (Luo et al., 2018), are key signaling events in the positive selection of GC B cells, but the loss of STIM proteins did not also affect their expressions (Fig. 3 D).

We determined the effect of STIM-mediated SOCE on Ag presentation by high-affinity B cells. To this end, we generated chimeric Ags consisting of NP and Eα-green fluorescent protein (GFP), which allowed us to measure Ag incorporation and presentation ability by detecting the expression of GFP and the Eα-MHC II complex (YA-e Ag) proteins, respectively (Pape et al., 2007). Given that the Ag presentation ability of GC B cells cannot be detected using this Ag in vivo (Inoue et al., 2017; Schwickert et al., 2011), we performed ex vivo experiments by incubating GC B cells sorted from NP-CGG–immunized mice with NP-EαGFP. Flow cytometric analysis results revealed that STIM-deficient B1-8^high^ Igλ^+^ GC B and control cells had similar percentages of GFP^+^YA-e^+^ cells (Fig. 3 E). Similar results were obtained when naive B cells were isolated from control and STIM-deficient B1-8^high^ mice (Fig. S2 C). These results suggest that STIM-mediated SOCE is unessential for BCR-mediated Ag presentation, which does not cause a reduction in the populations of GC B cells in the absence of STIM proteins.

### STIM-dependent SOCE suppresses GC B cell apoptosis

**Figure 4.**
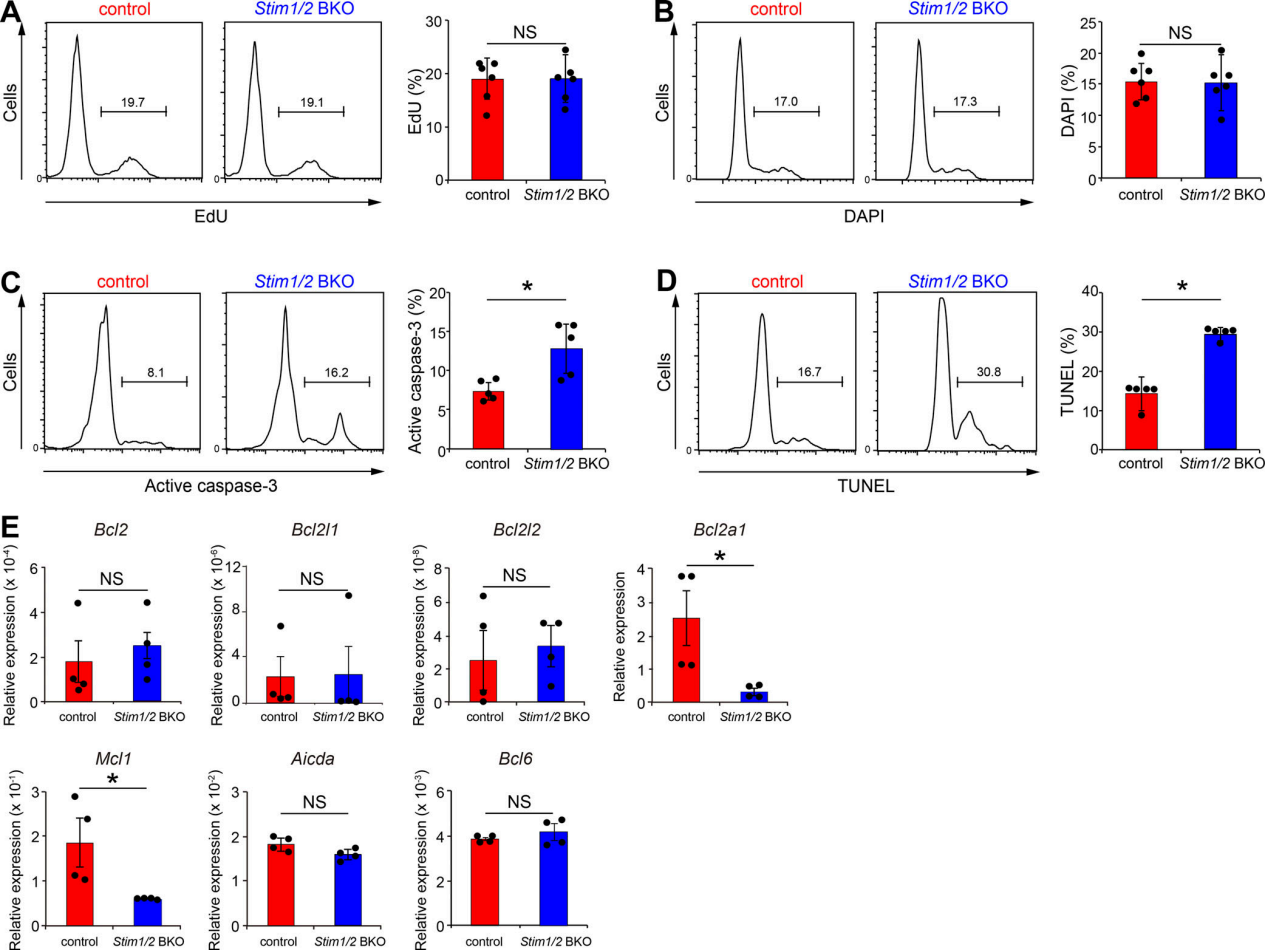
**STIM proteins suppress GC B cell apoptosis by the expressions of anti-apoptotic genes. (A–D)** Flow cytometry of CD45.1^+^ control B1-8^high^ and CD45.2^+^ *Stim1/2* BKO B1-8^high^ GC B cells with EdU incorporation (A), DNA content (B), active caspase-3 (C), and TUNEL staining of fragmented DNA (D) in CD45.1/2 wild-type mice transferred with equal number of CD45.1^+^ control B1-8^high^ and CD45.2^+^ *Stim1/2* BKO B1-8^high^ B cells, immunized with NP-CGG in alum for 7 days. Their percentages are shown on the right. Data are presented as mean ± SEM for six mice. Data shown are pooled from at least three independent experiments. NS, not significant. *, P < 0.05 versus control GC B cells (Student’s *t* test). **(E)** qRT-PCR analysis of GC B cells harvested from spleen of CD45.1/2 wild-type mice transferred with equal number of CD45.1^+^ control B1-8^high^ and CD45.2^+^ *Stim1/2* BKO B1-8^high^ B cells, immunized with NP-CGG in alum for 7 days. Data are normalized to the expression of GAPDH. Data are presented as mean ± SEM of four measurements. Data shown are pooled from two independent experiments. NS, not significant. *, P < 0.05 versus control GC B cells (Mann–Whitney *U* test).

Given that the number of GC B cells is regulated by the balance between their proliferation and apoptotic cell death (Green et al., 2011), we first examined whether STIM deficiency could lead to the reduction in GC B cell numbers through reduced cell growth. However, as demonstrated by 5-ethynyl-2′-deoxyuridine (EdU) and 4′,6′-diamidino-2-phenylindole (DAPI) staining, STIM-deficient B1-8^high^ GC B cells had similar proliferative responses to control cells in cotransferred mice with NP-CGG immunization (Fig. 4, A and B). Consequently, we considered whether B cell apoptosis was affected by examining the rates of apoptotic cell death in STIM-deficient GC B1-8^high^ B cells, based on the expression of active caspase-3, and DNA fragmentation by terminal deoxynucleotidyl transferase–mediated dUTP nick end labeling (TUNEL) assay. STIM protein deficiency promoted apoptosis in GC B cells (Fig. 4, C and D). Therefore, these results suggest that STIM-mediated signaling critically restrains apoptotic cell death in GC B cells; thus, STIM-deficient GC B cells were outcompeted by STIM-sufficient cells.

GC B cell survival is regulated by the expression of anti-apoptotic members of the Bcl-2 family (Strasser et al., 2009). Therefore, we detected the expression of anti-apoptotic genes associated with survival in GC B cells using quantitative reverse transcriptase-PCR (qRT-PCR). We confirmed the normal mRNA expression levels of GC-specific *Aicda* and *Bcl6* genes in STIM-deficient GC B cells (Fig. 4 E). Although the mRNA expression levels of *Bcl2*, *Bcl2l1*, and *Bcl2l2* were very low and unaffected by the loss of STIM proteins, STIM-deficient GC B cells showed significantly reduced mRNA expression of *Bcl2a1* and myeloid cell leukemia factor 1 (*Mcl1*) (Fig. 4 E). These results suggest that anti-apoptotic genes may regulate the survival of GC B cells in a SOCE-dependent manner.

### Essential role of STIM proteins in *Bcl2a1* expression

**Figure 5.**
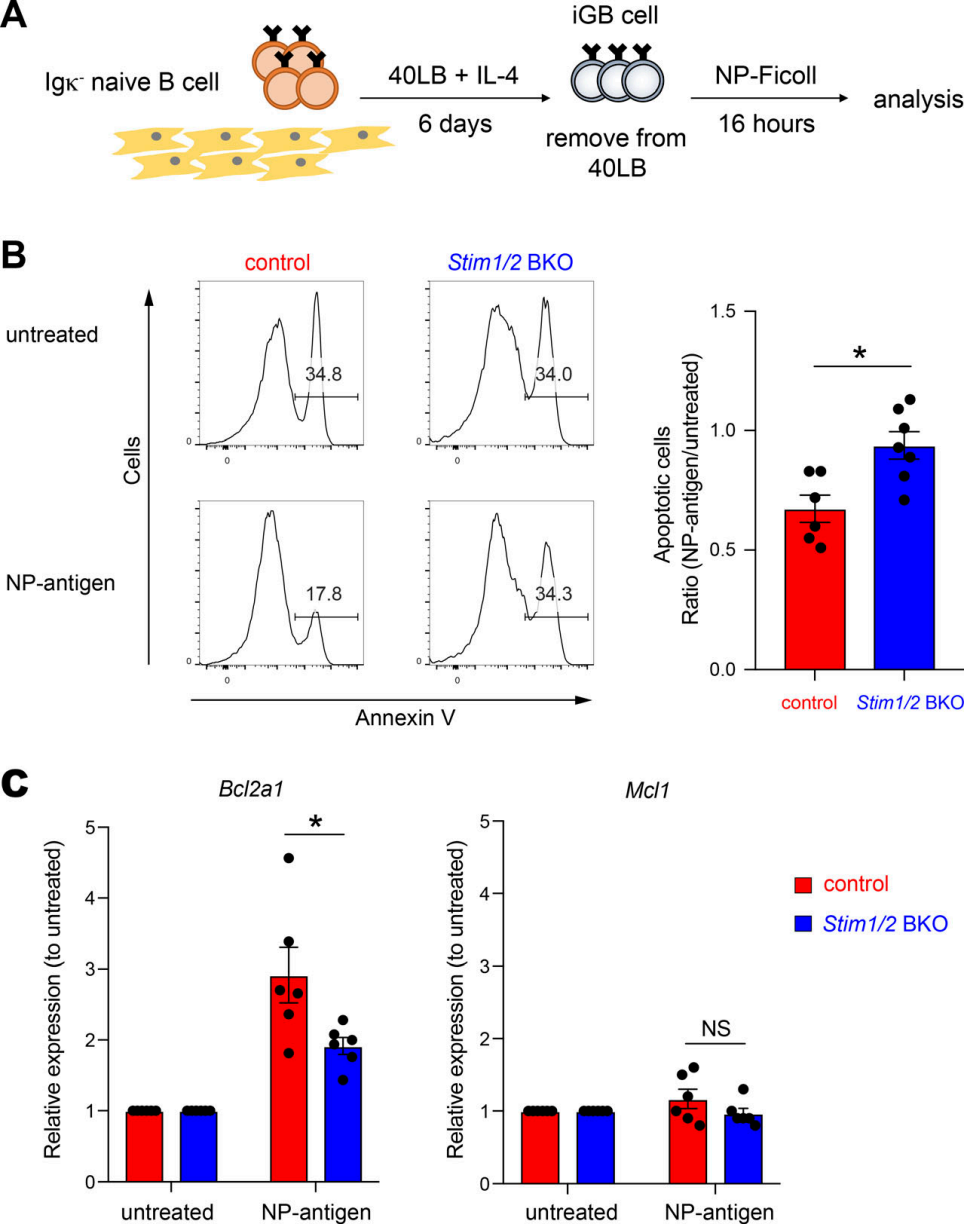
***Bcl2a1* expression in induced GC B cells depends on STIM proteins. (A)** Schematic of experimental workflow. Igκ-negative naive B cells from control B1-8^high^ and *Stim1/2* BKO B1-8^high^ mice using magnetic beads were cultured with 1 ng/ml IL-4 on 40LB feeder cells for 6 days. iGB cells were isolated from the feeders with the density gradient technique. Obtained Igκ-negative (Igλ-positive) iGB cells were stimulated with or without 4 µg/ml NP-Ficoll for 16 h. **(B)** Obtained Igκ-negative iGB cells from control B1-8^high^ and *Stim1/2* BKO B1-8^high^ B cells were stimulated with or without 4 µg/ml NP-Ficoll for 16 h. Representative flow cytometry is shown on the left. Percentages of Annexin V^+^ cells in iGB cells are shown as apoptotic cells. The ratio of apoptotic cells upon NP-Ficoll stimulation to untreated samples is shown on the right. Data are presented as mean ± SEM of six or seven measurements. Data are pooled from three independent experiments. Each experiment was performed with pooled B cells from at least five mice. *, P < 0.05 (Mann–Whitney *U* test). **(C)** qRT-PCR of mRNA encoding Bcl2a1 or Mcl1 in iGB cells after stimulation with NP-Ficoll, normalized to the expression of β-actin. Data are shown as relative expressions to unstimulated control. Data are presented as mean ± SEM of six measurements. Data shown are pooled from three independent experiments. Each experiment was performed with pooled B cells from at least five mice. NS, not significant. *, P < 0.05 (Student’s *t* test).

**Figure S3.**
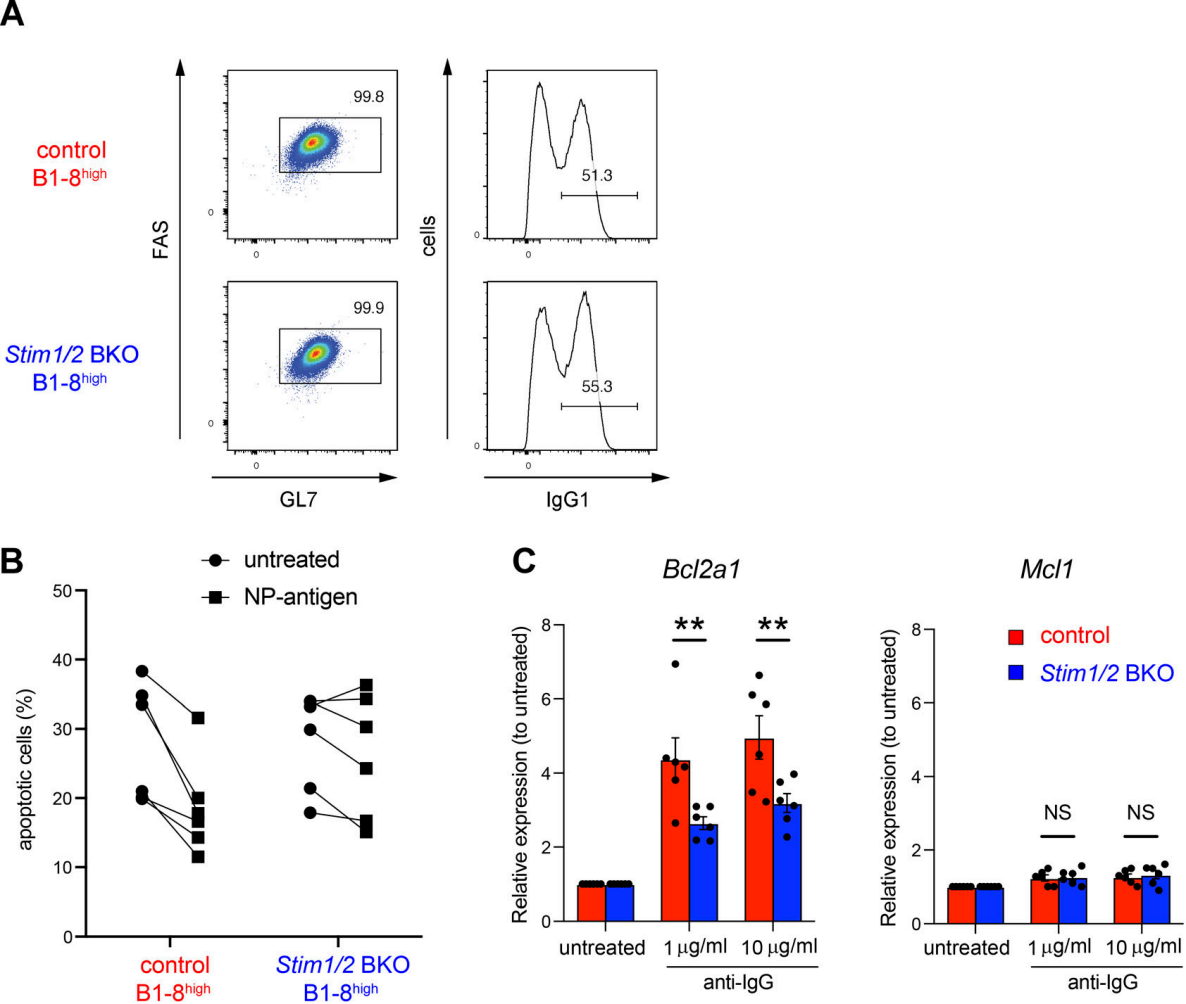
**STIM-mediated *Bcl2a1* expression in induced GC B cells decreases apoptosis.** Related to Fig. 5. **(A)** Representative flow cytometry of iGB cells obtained from control B1-8^high^ and *Stim1/2* BKO B1-8^high^ B cells. **(B)** Igκ-negative iGB cells obtained from control B1-8^high^ and *Stim1/2* BKO B1-8^high^ B cells were stimulated with or without 4 µg/ml NP-Ficoll for 16 h. Percentages of Annexin V^+^ cells in iGB cells are shown as apoptotic cells. **(C)** iGB cells obtained from control and *Stim1/2* BKO mice were stimulated with anti-IgG for 16 h. qRT-PCR of mRNA encoding Bcl2a1 or Mcl1 were assessed in iGB cells after stimulation with anti-IgG, normalized to the expression of β-actin. Data are shown as relative expression to unstimulated control. Data are presented as mean ± SEM of six measurements. Data are pooled from three independent experiments. NS, not significant. **, P < 0.01 (two-way ANOVA).

To directly assess the effects of STIM proteins on *Bcl2a1* and *Mcl1* expression, we used an in vitro GC B cell culture system (Fig. 5 A), which enabled the generation of a sufficient number of GC-like B cells to examine the signaling pathway (Nojima et al., 2011). First, naive Igκ^−^ (Igλ^+^) B cells were plated onto 40LB feeder cells expressing CD40L and BAFF plus IL-4, which drives exponential proliferation and generates GC-like B cells (iGB cells) (Haniuda and Kitamura, 2019). We detected >95% of STIM1/2-sufficient (control B1-8^high^) and -deficient (*Stim1/2* BKO B1-8^high^) B cells exhibiting GC-like features at day 6, suggesting that B cells could form GC B cells in the absence of STIM proteins in vitro (Fig. S3 A). To test the impact of BCR signaling, we stimulated Igλ^+^ iGB cells isolated from *Stim1/2* BKO B1-8^high^ and control B1-8^high^ mice with NP-Ficoll under feeder-free conditions. iGB cells appeared to die without receiving sufficient survival signals in the absence of 40LB feeder cells, whereas BCR stimulation promoted cell survival by suppressing apoptosis (Fig. 5 B and Fig. S3 B). Consistent with the in vivo results, *Stim1/2* BKO B1-8^high^ iGB cells stimulated with NP-Ficoll were more apoptotic than the control B1-8^high^ cells (Fig. 5 B and Fig. S3 B). qRT-PCR analysis of the control iGB cells showed that the mRNA expression of *Bcl2a1*, but not *Mcl1*, was markedly upregulated after Ag stimulation (Fig. 5 C), suggesting that *Mcl1* expression is independent of STIM proteins in vitro. In contrast, STIM-deficient iGB cells showed a defect in BCR-induced *Bcl2a1* expression, consistent with the in vivo experiments (Fig. 5 C and Fig. S3 C). These results suggest that STIM-dependent SOCE directly regulates the expression of *Bcl2a1* but not *Mcl1*.

### NFAT is required for STIM-mediated *Bcl2a1* expression

**Figure 6.**
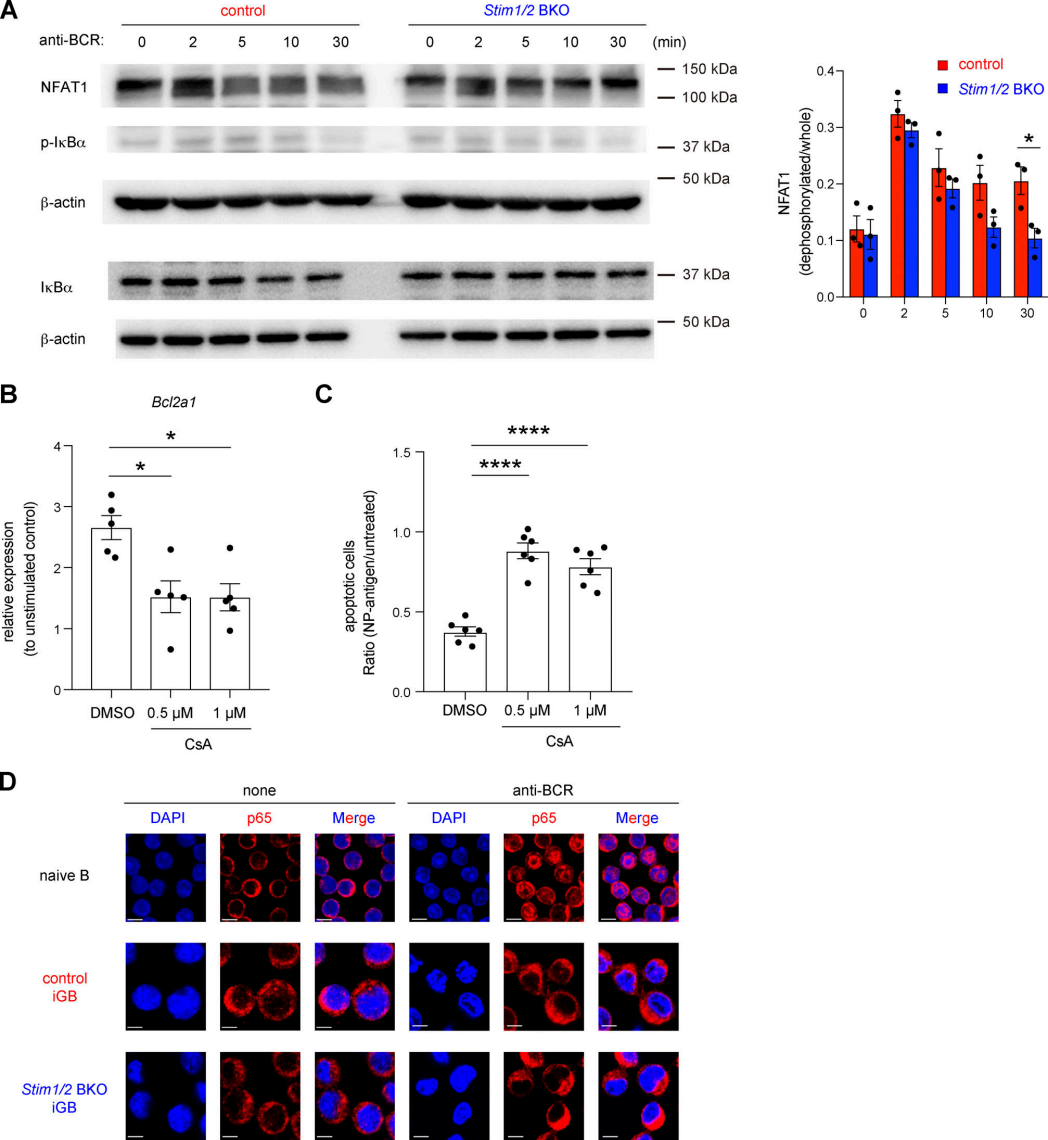
***Bcl2a1* expression is regulated by NFAT1. (A)** Naive B cells from control and *Stim1/2* BKO mice were cultured with 1 ng/ml IL-4 on 40LB feeder cells for 6 days before stimulation with 10 µg/ml anti-IgM and anti-IgG. Western blot analysis of NFAT1, phosphorylated IκBα (p-IκBα) and total IκBα were performed at the indicated time points. β-actin was used as a loading control. Representative data from three independent experiments are shown on the left. Densitometric analysis of NFAT1 pooled from three independent experiments is shown on the right. *, P < 0.05 (two-way ANOVA). **(B)** qRT-PCR of mRNA encoding Bcl2a1 in iGB cells after stimulation with anti-IgG in the presence or absence of CsA, normalized to the expression of β-actin. Data are shown as relative expressions to unstimulated control. Data are presented as mean ± SEM of five measurements. Data are pooled from two independent experiments. *, P < 0.05 (ordinary one-way ANOVA). **(C)** Obtained Igκ-negative iGB cells from B1-8^high^ B cells were stimulated with or without 4 µg/ml NP-Ficoll under the indicated concentration of CsA for 16 h. The ratio of apoptotic cells upon NP-Ficoll stimulation to untreated samples are shown. Data are presented as mean ± SEM of six measurements from two independent experiments. Each experiment was performed with pooled B cells from three mice. ****, P < 0.0001 (ordinary one-way ANOVA). **(D)** Naive B cells and obtained iGB cells from control and *Stim1/2* BKO mice were stimulated with or without 10 µg/ml anti-IgM and anti-IgG for 3 h. The nuclear localization of NF-κB was assessed by confocal microscopy. Scale bar shows 5 μm. Representative data are shown from two independent experiments. Source data are available for this figure: SourceData F6.

**Figure S4.**
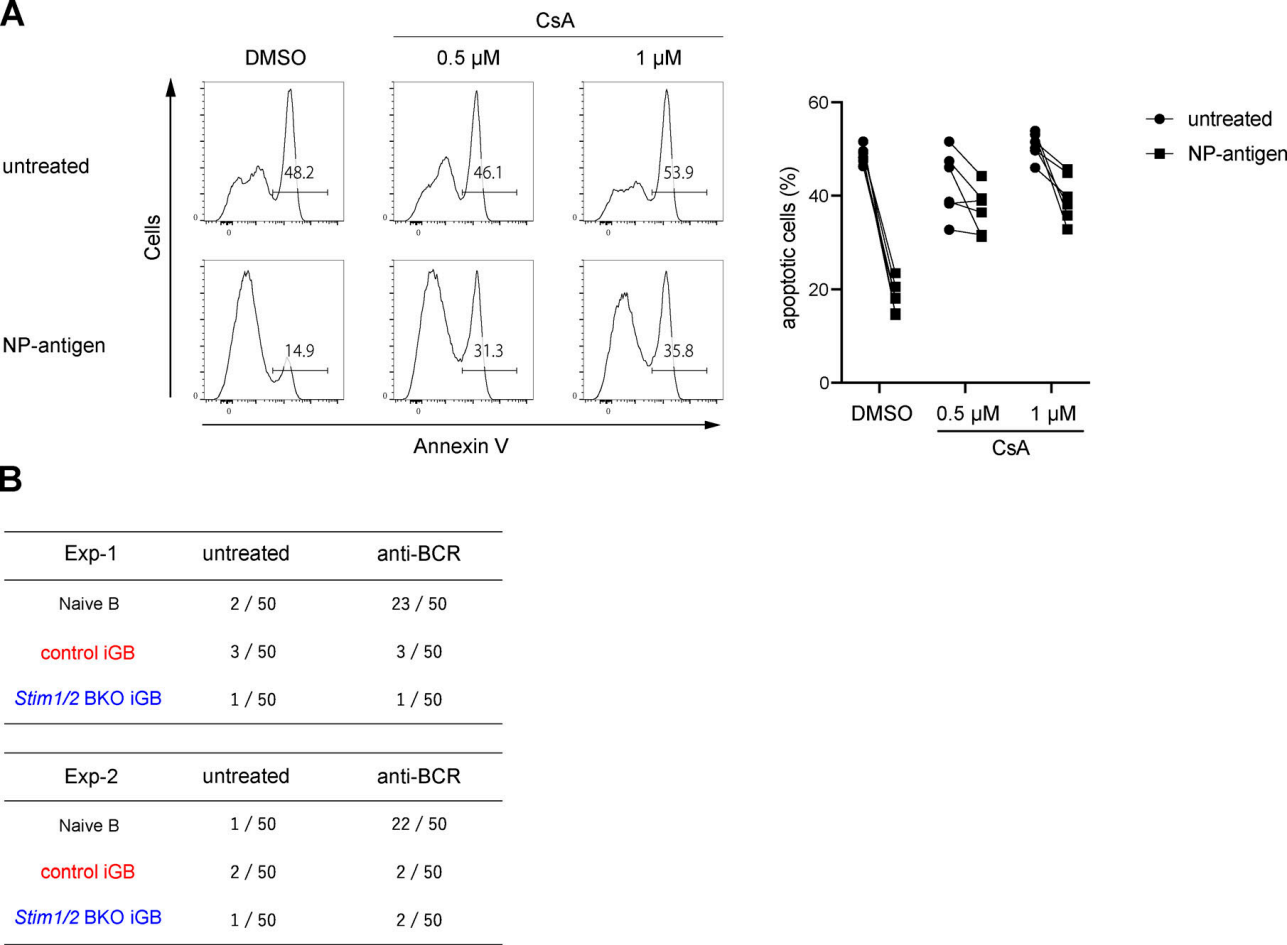
**GC B cell survival is regulated by the activation of calcineurin but not NF-κB.** Related to Fig. 6. **(A)** Obtained Igκ-negative iGB cells from B1-8^high^ B cells were stimulated with or without 4 µg/ml NP-Ficoll under the indicated concentration of CsA for 16 h. Representative flow cytometry of Annexin V^+^ cells is shown on the left. Percentages of apoptotic (Annexin V^+^) iGB cells are shown from two independent experiments on the right. **(B)** Naive B cells isolated from wild-type mice and iGB cells obtained from control and *Stim1/2* BKO mice were stimulated with or without 10 µg/ml anti-IgM and anti-IgG for 3 h. The nuclear localization of NF-κB was assessed by confocal microscopy. Data are presented as the number of cells with nuclear localization of p65 in 50 randomly selected cells.

Previous studies reported that the expression of Bcl2a1 is controlled by several signaling molecules, including NF-κB and NFAT, which are involved in downstream pathways of Ca^2+^ (Berry et al., 2018; Mandal et al., 2005; Xiao et al., 2021). To further assess the mechanism by which STIM-mediated SOCE regulates *Bcl2a1* expression, we first confirmed the activation status of NF-κB and NFAT in STIM1/2-sufficient and -deficient iGB cells. In STIM-deficient iGB cells, BCR-mediated transient activation of NFAT was comparable to that in control cells, but sustained activation of NFAT was attenuated more rapidly (Fig. 6 A). Importantly, calcineurin inhibitor cyclosporine A (CsA), which prevents NFAT activation in BCR-stimulated B cells (Baba and Kurosaki, 2011), suppressed the BCR-mediated *Bcl2a1* expression and survival (Fig. 6, B and C; and Fig. S4 A). On the other hand, NF-κB activation after BCR stimulation was not observed in either STIM1/2-sufficient or -deficient iGB cells, based on western blotting and nuclear localization of the p65 subunit of NF-κB (Fig. 6, A and D; and Fig. S4 B). These findings suggest that STIM-mediated NFAT activation is essential in BCR-dependent Bcl2a1 expression.

### Essential role of STIM-mediated *Bcl2a1* expression in GC B cell survival

**Figure 7.**
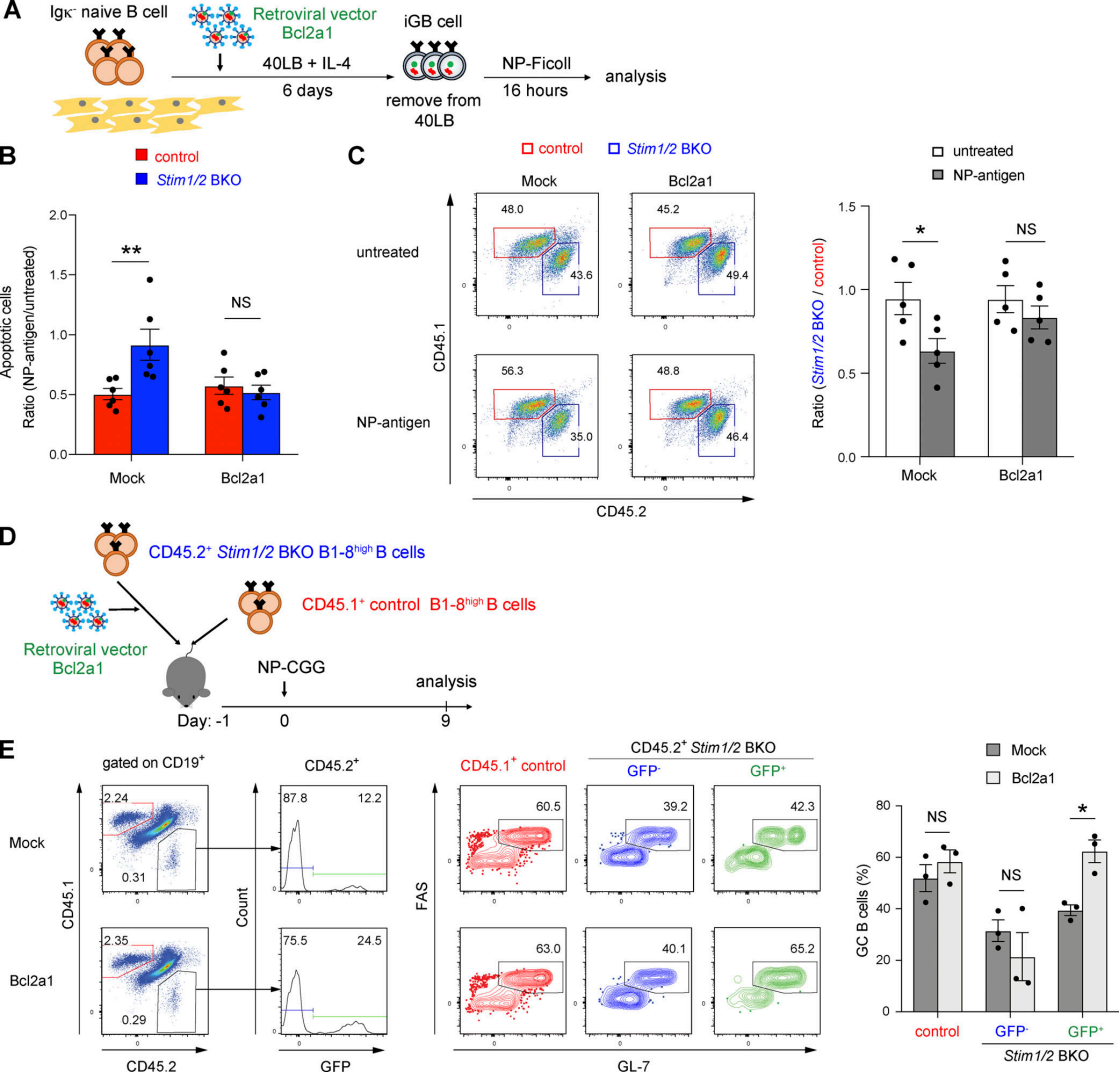
***Bcl2a1* transduction rescues apoptosis in STIM-deficient GC B cells. (A)** Schematic of retroviral transduction into iGB cells. Igκ-negative naive B cells from control B1-8^high^ and *Stim1/2* BKO B1-8^high^ mice were cultured with 1 ng/ml IL-4 on 40LB feeder cells. On day 2, cells were retrovirally transduced with *Bcl2a1*. On day 6, iGB cells were isolated from the feeders with density gradient technique, cultured with 4 µg/ml NP-Ficoll and analyzed after 16 h. **(B)** The ratios of apoptotic (Annexin V^+^) cells upon NP-Ficoll stimulation to untreated samples are shown. **(C)** Retrovirally *Bcl2a1*-transduced iGB cells from CD45.1^+^ control B1-8^high^ and CD45.2^+^ *Stim1/2*^+^ BKO B1-8^high^ B cells were mixed in a 1:1 ratio before co-culture for 16 h with or without 4 µg/ml NP-Ficoll. Representative flow cytometry of CD45.1^+^ or CD45.2^+^ cells gated on GFP^+^ cells are shown on the left. The ratios of *Stim1/2* BKO B1-8^high^ B cells to control B1-8^high^ B cells are shown on the right. **(D)** Schematic of experimental workflow. Equal numbers of CD45.2^+^ *Stim1/2* BKO B1-8^high^ B cells retrovirally transduced with *Bcl2a1* after prestimulation with anti-CD40 (1 μg/ml), IL-2 (10 ng/ml), IL-4 (10 ng/ml), and IL-5 (10 ng/ml) for 24 h and CD45.1^+^ control B1-8^high^ naive B cells were transferred into CD45.1/2 wild-type mice. The next day, the mice were immunized with NP-CGG in alum and analyzed after 9 days. **(E)** Representative flow cytometry. Frequency of GC B cells in the fractions of transferred CD45.1^+^ control B1-8^high^ B cells and non-infected or infected CD45.2^+^ *Stim1/2* BKO B1-8^high^ B cells on the left. The percentage of GC B cells in each fraction is shown on the right. CD45.1^+^ control B1-8^high^ B cells and non-infected or infected CD45.2^+^ *Stim1/ 2* BKO B1-8^high^ B cells are defined as CD19^+^CD45.1^+^CD45.2^−^ and CD19^+^CD45.1^−^CD45.2^+^GFP^−^ or CD19^+^CD45.1^−^CD45.1^+^GFP^+^ cells, respectively. GC B cells in each fraction are defined as FAS^+^GL7^+^ cells. **(B and C)** Data are presented as mean ± SEM of five or six measurements. Data are pooled from two independent experiments. Each experiment was performed with pooled B cells from at least three mice. NS, not significant. *, P <0.05, **, P <0.01 (two-way ANOVA). **(E)** Data are presented as mean ± SEM of three mice. Data are representative of two independent experiments. NS, not significant. *, P < 0.05 (two-way ANOVA).

**Figure S5.**
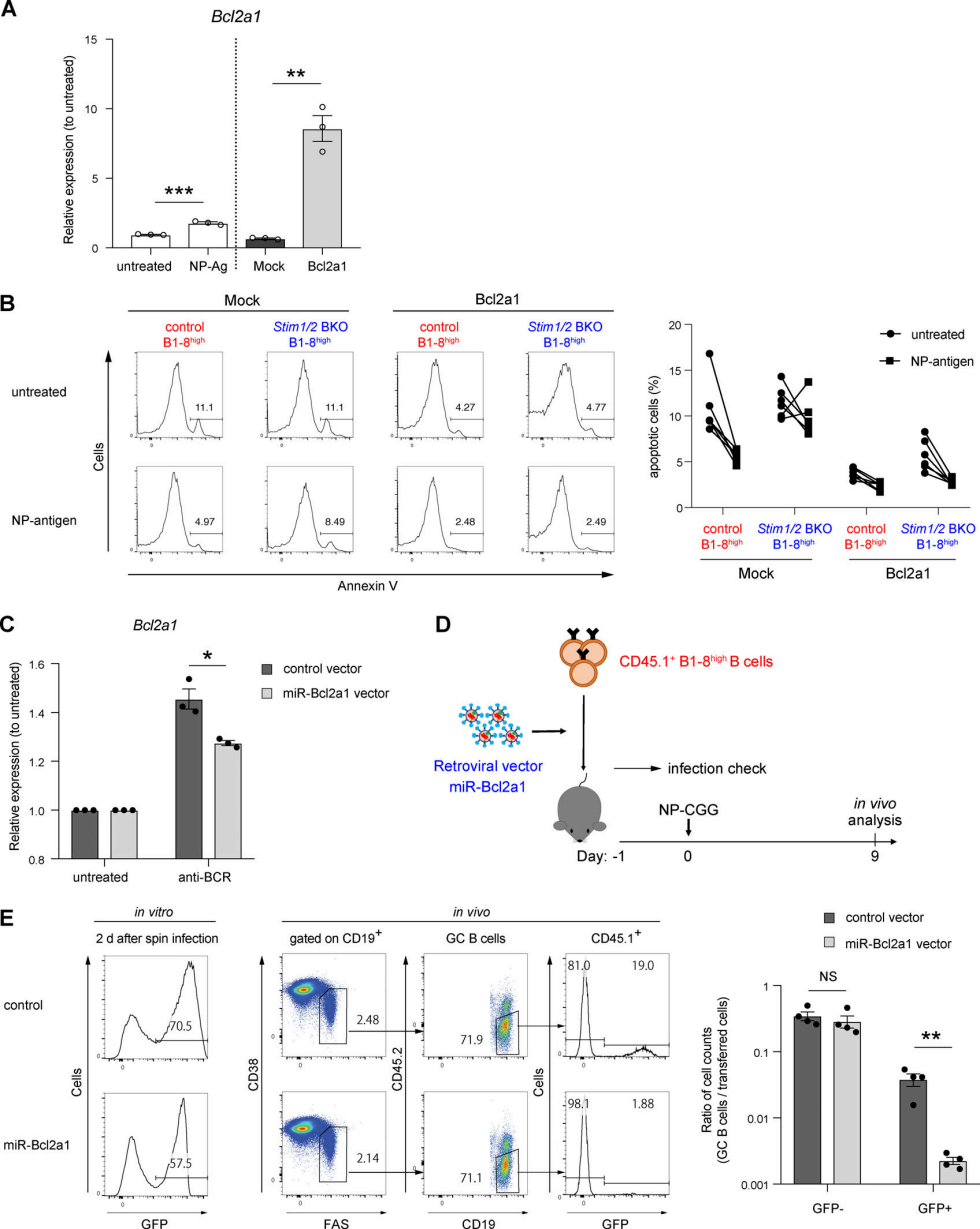
**Essential role of STIM-mediated *Bcl2a1* expression in GC B cell survival.** Related to Fig. 7. **(A)** qRT-PCR of mRNA encoding Bcl2a1 in iGB cells after stimulation with NP-Ficoll (left), or purified iGB cells after retroviral transduction with *Bcl2a1* (right). Data are normalized to the expression of β-actin. Data are presented as mean ± SEM of three measurements. Data are shown as relative expressions to unstimulated control. **, P < 0.01, ***, P < 0.001 (Student’s *t* test). **(B)** Retrovirally *Bcl2a1*-transduced iGB cells from control B1-8^high^ and *Stim1/2* BKO B1-8^high^ mice were stimulated for 16 h with or without 4 µg/ml NP-Ficoll. Representative flow cytometry of Annexin V^+^ cells is shown on the left. Percentages of apoptotic (Annexin V^+^) iGB cells are shown from two independent experiments on the right. **(C)** iGB cells retrovirally transduced with miR-Bcl2a1 were stimulated with anti-BCR for 2 h. The expression of mRNA encoding Bcl2a1 in purified GFP^+^ iGB cells was normalized to the expression of β-actin. Data are presented as mean ± SEM of three measurements. Data are shown as relative expressions to unstimulated control. *, P < 0.05 (Student’s *t* test). **(D)** Schematic of experimental workflow. CD45.1^+^ B1-8^high^ B cells retrovirally transduced with miR-Bcl2a1 after prestimulation with anti-CD40 (1 μg/ml), IL-2 (10 ng/ml), IL-4 (10 ng/ml), and IL-5 (10 ng/ml) for 24 h were transferred into CD45.1/2 wild-type mice. The next day, the mice were immunized with NP-CGG in alum, and analyzed after 9 days. **(E)** The percentage of retroviral transduction as GFP^+^ cells assessed in vitro is shown on the left. The percentage of transferred cells in GC B cells analyzed after 9 days of immunization is shown in the center as a representative flow cytometry. The transferred B cells transduced with and without retroviral vectors are represented as GFP^+^ and GFP^−^ cells, respectively. The ratio of the number of GC B cells after immunization to the number of transferred cells calculated based on the in vitro transduction frequency is shown on the right. Data are presented as mean ± SEM of four mice. Data are representative of two independent experiments. **, P < 0.01 (Student’s *t* test with Bonferroni correction).

To investigate whether apoptosis due to STIM deficiency was dependent on *Bcl2a1* expression, we retrovirally transduced *Bcl2a1* into STIM-deficient iGB cells (Fig. 7 A and Fig. S5 A). We found that apoptosis was reduced in *Stim1/2* BKO B1-8^high^ iGB cells transduced with *Bcl2a1* before stimulation with NP Ag (Fig. 7 B and Fig. S5 B). In an in vitro competitive setting, *Bcl2a1* transduction attenuated the rates of cell death caused by STIM deficiency (Fig. 7 C). These findings suggest that STIM-dependent Bcl2a1 expression may prevent GC B cell apoptosis.

To further assess the anti-apoptotic effect of Bcl2a1 in STIM-deficient GC B cells in vivo, we retrovirally transduced *Bcl2a1* into CD45.2^+^ *Stim1/2* BKO B1-8^high^ B cells and then cotransferred them with CD45.1^+^ control B1-8^high^ B cells into congenic CD45.1/2 recipient mice 1 day before immunization with NP-CGG (Fig. 7 D). Transduction with *Bcl2a1* significantly increased the frequency of GC B cells and resulted in greater enrichment in the GC compartment in the absence of STIM proteins compared with that in the vector control (Fig. 7 E). Conversely, silencing *Bcl2a1* expression by retroviral knockdown approach (Ottina et al., 2012) enhanced apoptosis in wild-type GC B cells in vivo (Fig. S5, C–E). These findings suggest that BCR-mediated Bcl2a1 expression is needed to prevent cell death during affinity maturation.

## Discussion

Our findings indicated that STIM-mediated SOCE regulated the positive selection and affinity maturation of GC B cells. Although STIM proteins are not essential for the proliferation of GC B cells, they regulated the survival of high-affinity B cells in GCs via the BCR-mediated expression of the anti-apoptotic *Bcl2a1* gene. This survival signal was in turn induced by STIM-dependent NFAT activation. These results provide evidence of the importance of T cell–independent BCR signaling in GC B cell survival and maintenance via STIM proteins.

In our Aicd-Cre/STIM KO chimera model, the loss of STIM proteins did not affect GC formation until day 7 after immunization, indicating that STIM-mediated SOCE is not involved in GC generation. However, at the later stage of GC formation (day 28), the Aicd-Cre/STIM KO B cells showed a decrease in GC B cell numbers and BCR W33L mutations. In the transfer model of B1-8^high^ B cells with W33L mutation, STIM proteins were also required for B cell selection and maintenance, suggesting the importance of STIM-mediated SOCE in high-affinity GC B cells. In a high-affinity B1-8^high^ B cell transfer experimental setting, STIM-deficient B cells are extensively outcompeted by control cells compared to that in Aicd-Cre BM chimera model. One possible explanation for this difference could be that STIM-mediated SOCE is activated in B cells with high-affinity BCR. Indeed, GC B cells in the BM chimera did not have the W33L mutation 7 days after immunization. At a later stage, control GC B cells exhibited the W33L mutation, while *Stim1*^*f/f*^*Stim2*^*f/f*^*Aicd*^*Cre*^ GC B cells had fewer mutations, indicating that the remaining cells with the *Stim1*^*f/f*^*Stim2*^*f/f*^*Aicd*^*Cre*^ genotype contain many low-affinity cells in which STIM proteins are less demanding. Another possibility is that in the transferred experimental setting, recipient-derived GC B cells with high-affinity BCR could compete with donor cells in significant numbers.

B cell selection and affinity maturation require Ag presentation to Tfh cells by GC B cells via MHC II (Vinuesa et al., 2016; Bannard and Cyster, 2017; Ise and Kurosaki, 2019; Victora and Nussenzweig, 2022). MHC II–sufficient B cells are preferentially mobilized to early GCs over MHC II–insufficient B cells until GCs are established (Yeh et al., 2018), suggesting that the density of the peptide–MHC II complex on GC B cells controls entry into the GC, but not B cell selection. Our study showed that STIM did not affect Ag presentation but played an important role in late, rather than early-stage GC formation. Given that BCR signaling augments GC B cell selection when Tfh cells are limited (Turner et al., 2018), high-affinity GC B cells in late GCs may be selected by receiving STIM-dependent survival signals after Ag recognition, making them more susceptible to help from Tfh cells.

In the currently prevailing model, affinity-based selection within GCs is primarily driven by CD40 signaling from Tfh cells to induce c-Myc expression via activation of NF-κB (Luo et al., 2018). These signaling pathways are not induced by BCR stimulation alone; however, when BCR and CD40 are simultaneously ligated, BCR crosslinking enhances GC B cell selection by synergistically inducing c-Myc expression via the Syk-PI3K-Akt-Foxo1 pathway (Dominguez-Sola et al., 2015; Luo et al., 2018; Sander et al., 2015). In addition to c-Myc, mTORC1 activation is also induced by the CD40-mediated PI3K-Akt axis and is important for positive selection via DZ B cell proliferation (Ersching et al., 2017). In the present study, however, STIM deficiency reduced GC B cell numbers but did not affect the DZ/LZ ratio, phosphorylation of Akt and S6 protein, and c-Myc expression, suggesting that Ca^2+^ signaling is not required for zone-specific GC maintenance. In addition, STIM had no effect on the proliferative response, indicating that STIM-deficient GC B cells may proliferate once they escape apoptosis after Ag recognition. Our model provides another role for BCR signaling in high-affinity GC B cells as STIM-mediated SOCE upregulates Bcl2a1 expression, allowing GC B cells to survive. Although the exact role of BCR signaling alone in GC selection was not clear, recent in vivo and ex vivo studies showed that GC B cells can signal through the BCR, although the pathway is modified in comparison to naive B cells (Mueller et al., 2015; Nowosad et al., 2016) and, of note, a study (published during revision of our work) reported the importance of positive selection mechanisms in which BCR signaling per se is required for the survival of GC B cells (Chen et al., 2023). Our findings also support the key role of BCR signaling in selection within GC and provide additional molecular mechanistic insight into this concept.

GC B cell survival is regulated by the expression of Bcl-2 anti-apoptotic members, such as Mcl1 and Bcl2 (Strasser et al., 2009; Vikstrom et al., 2010). Our findings showed that the SOCE-dependent survival of GC B cells is accompanied by the activation of *Bcl2a1* and *Mcl1* in vivo; however, along with previous studies (Berry et al., 2020), our in vitro study indicated that BCR stimulation induced the expression of *Bcl2a1*, but not *Mcl1* or *Bcl2*, in a STIM-dependent manner. Thus, when GC B cells acquire Ags from FDCs, the STIM-dependent expression of Bcl2a1 may promote the survival and the positive selection of GCs, which is consistent with the result of a previous report showing defective BCR-dependent B cell survival following in vivo knockdown of *Bcl2a1* (Sochalska et al., 2016). Although some studies have shown that the expression of Bcl2a1 is controlled by NF-κB in many cells, including naive B cells (Mandal et al., 2005; Vogler, 2012), we did not observe IκBα phosphorylation and p65 nuclear transport in iGB cells following BCR engagement, indicating that BCR signaling in GC B cells did not induce NF-κB activation (Luo et al., 2018). Instead, we observed a crucial role for NFAT in regulating the expression of *Bcl2a1* in iGB cells. In mice, all quadruplicate *Bcl2a1* genes harbor two consensus NFAT-binding sites within their promoters, which are recognized by NFATc1 and NFATc2 (Xiao et al., 2021). Treatment with CsA, which prevents NFAT activation, inhibited the upregulation of *Bcl2a1* upon BCR stimulation, indicating that STIM-mediated SOCE regulates GC B cell survival via NFAT-dependent Bcl2a1 expression. However, this BCR-mediated survival signal may be insufficient for long-term anti-apoptotic gene expression because Ag-stimulated GC B cells subsequently received strong survival signals predominantly mediated by CD40 from Tfh cells. Therefore, during the positive selection of GC B cells, the survival of B cells after Ag recognition may promote affinity maturation by increasing the probability of receiving Tfh cell assistance. Our findings indicated that Mcl1 was not directly targeted by STIM proteins upon BCR stimulation. Given that Mcl1 is involved in GC preselection, but not SHM (Vikstrom et al., 2010), the decreased number of GC B cells in the absence of STIM proteins may not be due to the reduction of Mcl1 expression. However, our data do not exclude the possibility that the STIM deficiency indirectly reduced Mcl1 expression through distinct pathways. It should be also noted that the BCR response was significantly stronger when GC B cells were stimulated ex vivo with a membrane-tethering Ag that mimicked the display on FDCs compared to when soluble Ags were used (Nowosad et al., 2016). Further studies will be needed to understand the implications of in vitro experiments using iGB cells stimulated with soluble Ags in GC B cells in vivo.

We previously showed that STIM BKO mice showed normal B cell antibody responses in vivo (Matsumoto et al., 2011), whereas STIM deficiency in GC B cells reduced survival rates under competitive conditions. This seems to be consistent with the fact that the GC responses of high- and low-affinity B cells (B1-8^high^ and B1-8^low^) were comparable during affinity-driven selection in GCs but, in a competitive setting in the same mice, B1-8^high^ GC B cells outcompeted B1-8^low^ cells (Shih et al., 2002a). Similar results were observed with MHC II^+/−^ haploinsufficient B cells, which exhibited a significant disadvantage over wild-type competitors in GC formation when in direct competition with MHC II^+/+^ B cells after immunization (Yeh et al., 2018). Based on these results, in vivo experiments in a competitive environment would be appropriate to investigate the intrinsic role of BCR affinity and Ag presentation in the GC reaction.

Collectively, our results demonstrated that the BCR-STIM axis regulates GC B cell selection. This finding highlights the previously overlooked importance of BCR signaling, independent of T cell assistance. STIM proteins might control survival after Ag recognition until receiving T cell assistance. The analysis of more precise signaling molecules will reveal how affinity-based GC B cell selection is regulated.

## Materials and methods

### Mice

C57BL/6 mice were purchased from CLEA Japan. *Aicd*^*Cre*/+^ (Kwon et al., 2008), B1-8^high^ (Shih et al., 2002b), *Mb1*^*Cre*/+^ (Hobeika et al., 2006), μMT (Kitamura et al., 1991), and *Stim1*^f/f^*Stim2*^f/f^*Mb1*^*Cre*/+^ (*Stim1/2* BKO) mice (Matsumoto et al., 2011) have been described previously. CD45.1^+^ mice were purchased from the Jackson Laboratory. We generated *Stim1*^f/f^*Stim2*^f/f^*Aicd*^*Cre*/+^ and *Stim1*^f/f^*Stim2*^f/f^*Mb1*^*Cre*/+^ (*Stim1/2* BKO) B1-8^high^ mice by crossing of *Stim1*^f/f^*Stim2*^f/f^ mice with *Aicd*^*Cre*/+^ mice and *Stim1*^f/f^*Stim2*^f/f^*Mb1*^*Cre*/+^ mice with B1-8^high^ mice, respectively. Mice were given intraperitoneally (i.p.) 100 μg of NP-CGG (Biosearch Technologies) in alum. Mice were bred and maintained under specific pathogen–free conditions and used at 6–16 wk. All studies and procedures were approved by the Animal Experiment Committee of Osaka University and Kyushu University. All animal experiments were conducted in accordance with the ARRIVE guidelines and the ethical guidelines of Osaka University and Kyushu University.

### Generation of mixed BM chimeras

Mixed BM chimeras were produced as described previously (Bannard et al., 2013). In brief, recipient μMT or CD45.1 wild-type mice received 800 cGy of x-ray irradiation. 1 day later, the recipients were reconstituted with a mixed inoculum of 50% CD45.1^+^CD45.2^+^*Aicd*^*Cre*/+^ BM cells plus 50% CD45.2^+^*Stim1*^f/f^*Stim2*^f/f^*Aicd*^*Cre*/+^ BM cells, or 50% CD45.1^+^CD45.2^+^*Stim1*^+/+^*Stim2*^+/+^*Mb1*^*cre*/+^ BM cells plus 50% CD45.2^+^*Stim1*^f/f^*Stim2*^f/f^*Mb1*^*cre*/+^ BM cells. Chimeric mice were left to fully reconstitute their lymphoid system for at least 8 wk before analysis or immunization.

### Flow cytometry

For flow cytometry, single-cell suspensions prepared from spleen were stained with the following fluorochrome-conjugated antibodies purchased from BD Biosciences, BioLegend, eBioscience and Cell Signaling Technology: fluorescein isothiocyanate (FITC)–conjugated anti-CD45.1 (A20), anti-CD45.2 (104), anti-IgG1 (A85-1), anti-MHCII (M5/114.15.2), anti-Igκ (RMK-45); phycoerythrin (PE)-conjugated anti-Akt (J1-223.371), anti-Bcl6 (BCL-DWN), anti-phospho-S6 (D57.2.2E), anti-CD40 (3/23), anti-CD45.2 (104), anti-CD83 (Michel-19), anti-CD274 (MIH5), anti-CXCR5 (2G8), anti-FAS (15A7), anti-ICOSL (HK5.3), anti-Igκ (187.1), anti-IL-4R (I015F8), anti-IL-21R (4A9), anti-IgG1 (A85-1); peridinin chlorophyll protein complex-cyanine 5.5 (PerCP5.5)–conjugated anti-B220 (RA-6B2), anti-CD86 (GL-1), anti-Igκ (187.1), anti-GL7 (GL7); PE-Cy7–conjugated anti-CD38 (90), anti-CD45.1 (A20), IgM (RMM-1); allophycocyanin (APC)-conjugated anti-B220 (RA-6B2), anti-CD19 (6D5), anti-Igλ (RML-42); Pacific blue–conjugated anti-B220 (RA-6B2), anti-CD45.1 (A20); Alexa Fluor 647–conjugated anti-GL7 (GL7); Fixable Viability Dye eFluor 780; Zombie Aqua; Brilliant Violet 421 (BV421)–conjugated anti-FAS (Jo2), anti-CD138 (281-2); Biotin-conjugated anti-CXCR4 (2B11) and anti-Eα52-68/I-A^b^ peptide (YA-e); Rabbit anti-c-Myc (D84C12); and (4-hydroxy-5-iodo-3-nitrophenyl) acetyl (NIP)-BSA conjugated with APC was used for detection of (4-hydroxy-3-nitrophenyl) acetyl-specific B cells. For EdU experiments, mice received a single i.p. injection of 1 mg EdU (RRID:SCR_008988; Sigma-Aldrich) 30 min prior to euthanasia. EdU staining was performed as per the manufacturer’s guidelines (BD Pharmingen). For cell-cycle analysis, cells were stained for GC markers and then fixed and permeabilized with BD cytofix/cytoperm buffer. DAPI (1 μg/ml; Sigma-Aldrich) was added to FACS tubes before FACS analysis. For detection of active caspase-3 and TUNEL assays (terminal deoxynucleotidyl transferase-mediated dUTP nick end-labeling), 1 × 10^7^ cells in 10 cm cell culture dishes were incubated for 4 h at 37°C in RPMI-1640 medium containing 0.5% (vol/vol) FCS. Cells were then stained for DNA fragmentation with an active caspase-3 apoptosis kit and APO-BRDU apoptosis detection kit according to the manufacturer’s protocol (BD Biosciences).

For intracellular staining, splenocytes or iGB cells were fixed and permeabilized with Foxp3 Staining Buffer Set (eBioscience) before intracellular staining with FITC-conjugated anti-Bcl6, PE-conjugated anti-pS6, or Rabbit anti-c-Myc and following PE-conjugated secondary antibody and then analyzed on FACSCantoII (RRID:SCR_018055; BD Biosciences) or Cytoflex (RRID:SCR_019627; Beckman Coulter). For Akt detection, splenocytes were fixed with BD Phosflow Fix Buffer I (BD Biosciences), permeabilized in BD Phosflow Perm Buffer III (BD Biosciences), and then stained with PE-conjugated anti-Akt antibody.

### Sorting and isolation of B cells and adoptive transfer

Cell sorting was done on BD FACS Aria II (RRID:SCR_018091) or BD FACSMelody Cell Sorter (RRID:SCR_023209). For isolation of single IgG1^+^NIP^+^CD38^low^B220^+^ clones, splenocytes of BM chimeric mice 7, 14, and 28 days after NP-CGG immunization were stained with APC-NIP, FITC-anti-IgG1, PB-anti-CD45.1, PE-anti-CD45.2, PerCP5.5-anti-B220, and PECy7-anti-CD38. For isolation of FAS^+^GL7^+^B220^+^ cells, splenocytes were stained with APC-anti-GL7, FITC-anti-CD45.2, PB-anti-CD45.1, PE-anti-FAS, and PerCP5.5-anti-B220.

For B cell isolation, splenic B cells were purified by negative selection of CD43^+^ cells with anti-CD43 magnetic beads (RRID:SCR_008984; Miltenyi Biotec). The enriched B cell population was >95% positive for B220 or CD19 staining. An equal number of B cells (2–5 × 10^6^ cells) from the spleen of CD45.1^+^ control B1-8^high^ and CD45.2^+^ *Stim1/2* BKO B1-8^high^ mice was transferred intravenously into CD45.1/2 wild-type mice 24 h before immunization with NP-CGG.

### SHM assay

Sequence analysis of NP-specific GC B cells or plasma cells was performed as described previously (Muramatsu et al., 2000). In brief, single NP-specific IgG1^+^ GC B cells or NP-specific IgG1^+^ plasma cells (NIP^+^CD138^+^TACI^+^IgG1^+^) were directly sorted into a 96-well PCR plate. RT-PCR mixture (SuperScript One-step High Fidelity kit; Invitrogen) was added and subjected to nested PCR. The first PCR product was used for the second round of PCR using Platinum Pfx DNA polymerase (Invitrogen). The PCR products were directly sequenced using Cγ1 internal antisense primers.

### Ca^2+^ measurement

Cytosolic Ca^2+^ concentrations were measured as described previously (Baba et al., 2006). In brief, splenocytes were loaded with indo-1 acetoxymethylester (Indo-1 AM) and Pluronic F-127 (Invitrogen) and stained with antibodies to B220 and Igκ. Cells were stimulated with 10 μg/ml NP-Ficoll (Biosearch Technologies). Changes in fluorescence intensity were monitored on an LSR flow cytometer (BD Biosciences).

### qRT-PCR analysis

Total RNA was purified with the TRIzol reagent (Invitrogen) and subjected to cDNA synthesis using SuperScript first-strand synthesis system (Invitrogen) according to the manufacturer’s instructions. The following primer pairs were used for GC B cells or iGB cells: sense primer 5′-TAG​TGC​CAC​CTC​CTG​CTC​ACT-3′ and antisense primer 5′-CAACAATTCCACGTGGCAGCC-3′(Aicda); sense primer 5′-GAG​CGT​CAA​CAG​GGA​GAT​G-3′ and antisense primer 5′-CAG​AGA​CAG​CCA​GGA​GAA​ATC-3′ (Bcl2); sense primer 5′-TGA​ATA​ACA​CAG​GAG​AAT​GGA​TAC​G-3′ and antisense primer 5′-GAA​ATG​CCA​AGT​GCT​GAT​AAC​C-3′ or 5′-TTC​CCA​GAT​CTG​TCC​TGT​CA-3′ (only in Fig. S5A) (Bcl2a1); sense primer 5′-GGA​AAG​CGT​AGA​CAA​GGA​GAT​G-3′ and antisense primer 5′-CCC​GTA​GAG​ATC​CAC​AAA​AGT​G-3′ (Bcl2l1); sense primer 5′-CGT​CTT​GTG​GCA​TTC​TTT​GTC-3′ and antisense primer 5′-TCC​CCG​TAT​AGA​GCT​GTG​AA-3′ (Bcl2l2); sense primer 5′-GCC​CAC​GTT​CCC​GGA​GGA​GA-3′ and antisense primer 5′-CGT​CTG​CAG​CGT​GTG​CCT​CT-3′ (Bcl6); sense primer 5′-TTC​ACC​ACC​ATG​GAG​AAG​GCC​G-3′ and antisense primer 5′- GGC​ATG​GAC​TGT​GGT​CAT​GA-3′ (GAPDH); sense primer 5′- GCT​CTT​TTC​CAG​CCT​TC-3′ and antisense primer 5′- CGG​ATG​TCA​ACG​TCA​CA-3′ (β-actin). The Mcl-1 primer assay (mM_Mcl1_1_SG; Quantitect) was from Qiagen.

### Ag presentation assay

NP-EαGFP proteins were generated by coupling NP-succinimide ester (NP-Osu; Biosearch Technology) to the EαGFP proteins, as reported previously (Ise et al., 2014). B cells isolated from spleen of NP-immunized mice adoptively transferred with CD45.1^+^ control B1-8^high^ and CD45.2^+^ *Stim1/2* BKO B1-8^high^ B cells were incubated without or with 0.1 mg/ml EαGFP or NP-EαGFP for 1 h, and then the presentation of Eα52-68/I-A^b^ on Igκ^−^Fas^+^GL7^+^ GC B cells was detected with biotin-conjugated anti-Y-Ae antibody followed by incubation with BV650-conjugated streptavidin (eBioscience).

### Cells and in vitro culture assay

40LB cells were cultured and maintained at 37°C with 5% CO_2_ in D-MEM (RRID:SCR_013651; Wako) containing 10% (vol/vol) FCS and penicillin/streptomycin (RRID:SCR_013519; Nacalai Tesque), as reported previously (Haniuda and Kitamura, 2019). Splenic B cells (5 × 10^5^ cells) were grown on confluent 40LB feeder cells in a 10-cm dish (about 4 × 10^6^ cells). 40LB cells were seeded in a 10-cm dish the day before and pre-treated with mitomycin (RRID:SCR_006140; R&D Systems) on the day. Cells were cultured at 37°C with 5% CO_2_ in RPMI-1640 medium containing 10% (vol/vol) FCS, HEPES, *L*-glutamine, penicillin/streptomycin, and sodium pyruvate (Nacalai Tesque). IL-4 (1 ng/ml; R&D Systems) was added to the culture media. Cells were cultured on the same feeders for 6 days. After a 6-day culture in the presence of 40LB cells, the B cells were isolated from the feeders using the percoll density gradient method (RRID:SCR_023581; Cytiva). B cells were then cultured for a further 16 h in RPMI-1640 (as described above) with or without 4 µg/ml NP-Ficoll (Biosearch Technologies), 1 μg/ml anti-mouse IgG F(ab′)^2^ (Cat# 115-006-071, RRID:AB_2338472; Jackson ImmunoResearch Labs). Where indicated, the following reagents were added into in vitro cultures: 0.5–1 µM Cyclosporin A (Calbiochem). For cell apoptosis assays, iGB cells were stained with APC Annexin V in 1× binding buffer according to the manufacturer’s protocol (BD Biosciences). Total RNA was purified with the RNeasy Micro Kit (Qiagen) from iGB cells and subjected to cDNA synthesis using ReverTra Ace qPCR RT Master Mix with gDNA Remover (TOYOBO) according to the manufacturer’s instructions.

### Retroviral transduction

To generate a *Bcl2a1* retroviral expression vector, a cDNA corresponding to *Bcl2a1* obtained from mouse splenocytes by PCR amplification was cloned into the pMX-IRES-GFP retroviral vector. The resulting retroviral vector (pMX-Bcl2a1-IRES-GFP) and a GFP-alone control vector (pMX-IRES-GFP) were transfected into PLAT-E cells with FuGENE HD (Roche Diagnostics). At 24 h after transfection, the medium was changed and the cells were cultured for an additional 48 h. To express *Bcl2a1* in STIM-deficient B cells in vivo, splenic B cells were purified from *Stim1/2* BKO B1-8^high^ mice (CD45.1^−^CD45.2^+^) and then cultured with anti-CD40 (1 μg/ml), mIL-2 (10 ng/ml), mIL-4 (10 ng/ml), and mIL-5 (10 ng/ml) for 24 h in vitro. Cells underwent “spin infection” for 2 h at 32°C (800 *g*) after virus supernatant and polybrene (6 or 8 μg/ml) were added, and the plates were incubated at 37°C for 1 h. After washing the cells with PBS, equal numbers (1∼5 × 10^6^) of the infected *Stim1/2* BKO B1-8^high^ B cells (CD45.1^−^CD45.2^+^) and non-infected control B1-8^high^ (CD45.1^+^CD45.2^−^) B cells were transferred intravenously into CD45.1^+^CD45.2^+^ wild-type mice. 1 day after cell transfer, the mice were immunized i.p. with 100 μg of NP-CGG in alum on day 0 and analyzed on day 9.

For retroviral transduction of iGB cells, 1.5 × 10^5^ splenic B cells were grown on confluent 40LB feeder cells in each well of a six-well plate. 2 days after transduction, iGB cells underwent spin infection for 2 h at 32°C (800 *g*) after virus supernatant and polybrene (6 μg/ml) were added. After washing the cells, spin-infected iGB cells were cultured on the same feeders with fresh media for a further 4 days.

To generate a *Bcl2a1* silencing vector, the following oligonucleotide miR-Bcl2a1 was subcloned into pMYs-GFP vector: 5′-gtc​gac​aag​gta​tat​tgc​tgt​tga​cag​tga​gcg​cCC​ATA​GAT​ACC​GCC​AGA​ATA​ATA​gtg​aag​cca​cag​atg​TAT​TAT​TCT​GGC​GGT​ATC​TAT​GGa​tgc​cta​ctg​cct​cgg​cgg​ccg​c-3′ (sequence targeting *Bcl2a1* mRNA in capital letters) (Ottina et al., 2012). The resulting retroviral vector (pMYs-GFP-miR-Bcl2a1) and micro-RNA containing control vector (pMYs-GFP-miRNA) were transduced into splenic B cells from B1-8^high^ mice (CD45.1^+^CD45.2^−^) by spin infection as described above. The infected B1-8^high^ B cells (5 × 10^6^ cells) were intravenously transferred into CD45.1^+^CD45.2^+^ wild-type mice. 1 day after cell transplantation, the mice were i.p. immunized with 100 μg NP-CGG in alum (day 0) and analyzed on day 9.

### iGB cell stimulation for western blotting analysis and confocal microscopy analysis

Isolated iGB cells (1 × 10^6^ cells/ml) from 40LB feeder cells were stimulated with 10 μg/ml anti-IgM F(ab′)^2^ (Cat# 115-006-020, RRID:AB_2338469; Jackson ImmunoResearch Labs) and 10 μg/ml anti-mouse IgG F(ab′)^2^ (Cat# 115-006-071, RRID:AB_2338472; Jackson ImmunoResearch Labs) for indicated times and then lysed in lysis buffer containing 10 mM Tris-HCl (pH 7.4), 150 mM NaCl, 1% (vol/vol) Triton X-100, 0.5 mM EDTA plus protease, and phosphatase inhibitor cocktails (Nacalai Tesque). Samples were transferred to polyvinyldifluoride membranes by electrophoresis and antibodies against phospho-IκBα (Ser32/36) (5A5) (Cat# 9246, RRID:AB_2267145; Cell Signaling Technology), IκBα (44D4) (Cat# 4812, RRID:AB_10694416; Cell Signaling Technology), NFAT1 (D43B1) (Cat# 5861, RRID:AB_10834808; Cell Signaling Technology), and β-actin (C4) (Cat# sc-47778, RRID:AB_626632; Santa Cruz Biotechnology). For confocal microscopy, isolated iGB cells (as described above) were stimulated with 10 μg/ml anti-IgM F(ab′)^2^ (Jackson ImmunoResearch Laboratories) and 10 μg/ml anti-mouse IgG F(ab′)^2^ (Jackson ImmunoResearch Laboratories) for 3 h, and then were adhered to cover glasses precoated with Cell-Tak (Discovery Labware Inc.). The cellular specimens were fixed with ice-cold 100% methanol and then were rinsed and proceeded with immunostaining. For immunostaining, the following reagents were used: anti-NF-κB p65 (D14E12) (Cat# 8242, RRID:AB_10859369; Cell Signaling Technology) and DAPI (BioLegend). The specimens were analyzed with LSM700 (RRID:SCR_017377; Zeiss).

### Statistical analysis

We performed statistical evaluation using Prism software (RRID:SCR_002798; GraphPad). A two-tailed, unpaired Student’s *t* test was applied for the statistical comparison of the two groups. In case of unequal variance, *t* test with Welch’s correction was used. Comparisons of two nonparametric datasets were done by the Mann–Whitney *U* test. Analysis of variance (ANOVA) was applied for statistical comparison between multiple groups. A P value of <0.05 was considered statistically significant.

### Online supplemental material

Fig. S1 shows the frequency of CD45.1^+^CD45.2^+^*Aicd*^*Cre*/+^ and CD45.2^+^*Stim1*^f/f^*Stim2*^f/f^*Aicd*^*Cre*/+^ cells in total B, follicular B, and NP-specific GC B cells in the spleen of mixed BM chimeric mice immunized with NP-CGG in alum. Fig. S1 also contains the frequency of W33L^+^ clones among single NP-specific IgG1^+^ plasma cells in mixed BM chimeric mice immunized with NP-CGG. Fig. S2 shows flow cytometry data of control B1-8^high^ and *Stim1/2* BKO B1-8^high^ splenocytes, including the information on Igλ light chain usage, IgM expression, and in vitro Ag-presenting ability. Fig. S3 shows flow cytometry data of iGB cells obtained from control B1-8^high^ and *Stim1/2* BKO B1-8^high^ B cells. Fig. S3 also contains all sample data underlying Fig. 5 B and qRT-PCR data for *Bcl2a1* or *Mcl1* mRNA transcripts in control and Stim1/2 BKO iGB cells stimulated with anti-IgG. Fig. S4 contains all sample data underlying Fig. 6 C and the information about the number of cells with nuclear localization of p65 shown in Fig. 6 D. Fig. S5 shows qRT-PCR of mRNA encoding Bcl2a1 after retroviral transduction of *Bcl2a1* and all sample data underlying Fig. 7 B. Fig. S5 also contains the knockdown efficiency of miR-Bcl2a1 in vitro based on qRT-PCR, a schematic of the experimental workflow of an in vivo *Bcl2a1* knockdown study, and its result.

## Supplementary Material

SourceData F6 contains original blots for Fig. 6.

## Data Availability

The data underlying all figures can be found in the paper or the online supplemental material.
