# Supplementary material for: STIM-mediated calcium influx regulates maintenance and selection of germinal center B cells
Source: J Exp Med. 2023 Oct 30;221(1):e20222178. doi: 10.1084/jem.20222178 (PMC10615893; doi:10.1084/jem.20222178)
Supplement: SourceData F6 — contains original blots for Fig. 6. [file JEM_20222178_SourceDataF6.pdf]

Source data Figure 6A

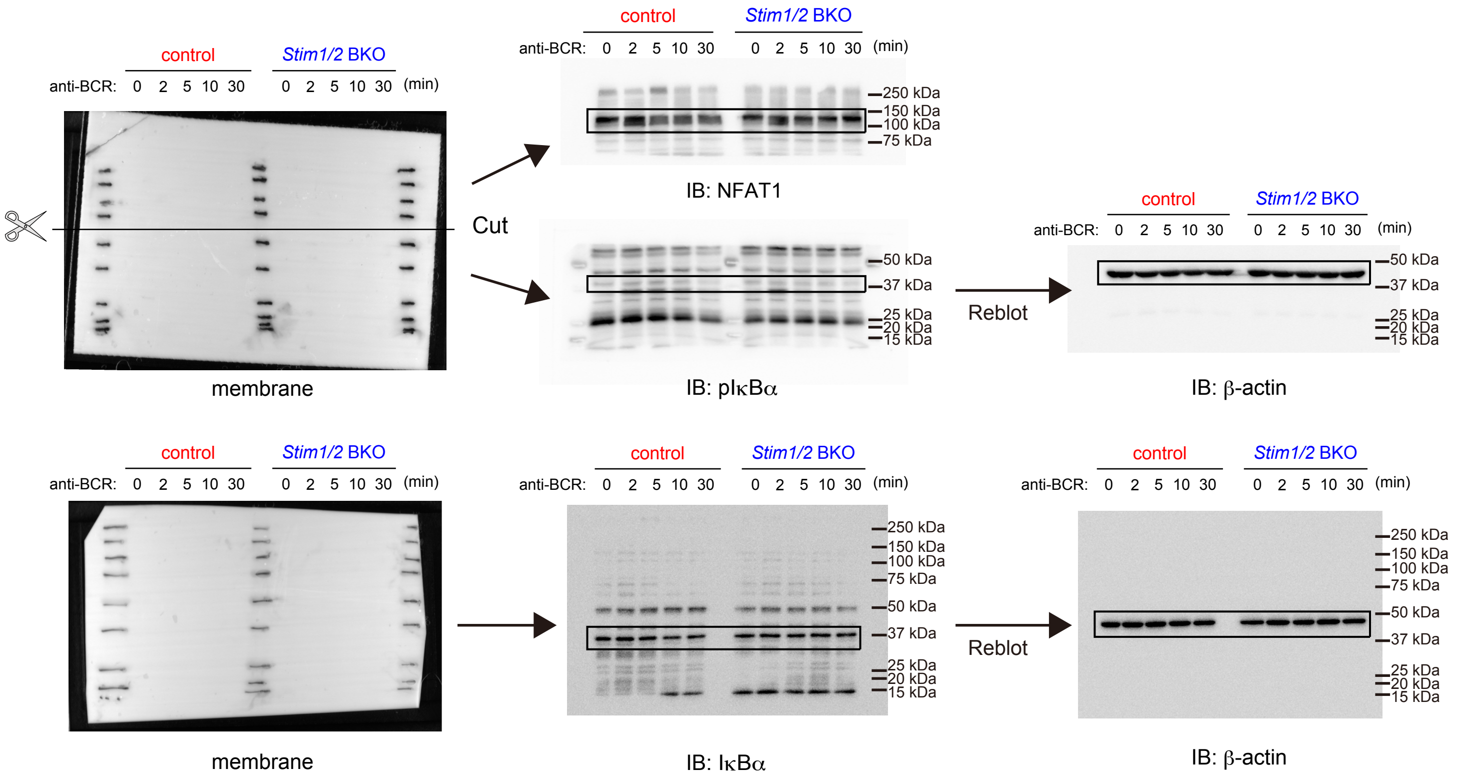

Source data Fig.6A:

Obtained iGB cells from control and Stim1/2 BKO were stimulated with 10 μg/ml anti-IgM and anti-IgG to indicated time points.

Full Western blotting of NFAT1 (inactive form 140 kDa), phosphorylated IκBα (p-IκBα) (40 kDa) and total IκBα (39 kDa) were performed.

β-actin (43 kDa) was assessed after reblotting the same membrane as a loading control.

The area cropped with a box were shown Figure 6A.
